# Indirect experiential grounding: semantic similarity of abstract scientific concepts is reflected in activity patterns in visual and motor cortex

**DOI:** 10.1038/s41598-025-32189-2

**Published:** 2025-12-12

**Authors:** Martin Ulrich, Marcel Harpaintner, Natalie M. Trumpp, Alexander Berger, Fritz Günther, Markus Kiefer

**Affiliations:** 1 Department of Psychiatry, Ulm University, Ulm, Germany; 2 Department of Psychology, Humboldt University, Berlin, Germany

**Keywords:** Representational similarity analysis, RSA, Abstract scientific concepts, Grounded cognition, Expertise, Mentalizing, Science education, Neuroscience, Psychology, Psychology

## Abstract

Grounded cognition theories propose that interactions with situations establish experiential memory traces that constitute conceptual meaning. However, as abstract scientific concepts are frequently learned through language, a proposed indirect grounding mechanism postulates that modal representations are extrapolated from distributional language-based representations, which are themselves mapped onto existing modal representations. Using functional magnetic resonance imaging (*n* = 51), we tested the mapping of distributed language representations on modal representations by asking whether semantic similarity of distributional language representations of scientific concepts corresponds to the similarity of neural activation patterns in modal brain systems. Semantic similarity based on both distributed language representations and experiential features was reflected in the multi-voxel activity pattern within occipital and fronto-parietal cortex, overlapping with activation induced by real visual perception and hand action, in addition to multimodal areas outside the brain regions sensitive to perception or action. Although its functional relevance for extrapolating modal representations remains to be determined, this mapping between language and the visuo-motor system is a fundamental element of the indirect grounding mechanism. This mechanism provides sensory-motor experience for the unseen and enriches knowledge for concepts learned exclusively from language.

**Supplementary Information:**

The online version contains supplementary material available at 10.1038/s41598-025-32189-2.

## Introduction

Concepts serve as the fundamental building blocks of cognition because they provide the knowledge structure for thinking and establish word meaning^1^. Humans possess the remarkable ability to retain conceptual knowledge about entities that surpass sensory perception^2^ such as everyday concepts like “expectation”, but also scientific constructs like “gravitation” or “frequency”. In the realm of psychological science, many constructs naturally fall under the umbrella of abstract concepts, referring to various facets of mental phenomena inaccessible to direct perception, such as “conditioning” or “habituation”.

The functional and neuroanatomical organization of human long-term memory for abstract concepts, including scientific ones, lies at the heart of an ongoing debate. Concepts have traditionally been viewed as amodal mental representations, distinct from the experiential modal brain systems associated with perception, action, and introspection (e.g.^3–5^). These amodal conceptual representations are assumed to be coded in the heteromodal association cortex, particularly within regions such as anterior temporal^6^, posterior temporal^7^ or inferior parietal^8^ cortex, often referred to as semantic hubs^9^. Although hub regions such as the anterior temporal or parietal cortex are frequently considered to hold domain-general amodal conceptual representations^8^, observations of category-specific deficits after lesions in these regions point to a possible category-specific function of these hubs^10–14^. Furthermore, particularly abstract concepts are assumed to rely on verbal representations^2^ stored in language regions of the brain^15^. More recent distributional models of semantics suggest that speakers form meaning representations from co-occurrence patterns of words in natural language^16–21^. As words with similar meanings tend to be used in similar linguistic contexts^22^, these co-occurrence patterns of words in natural language establish a semantic similarity space, which defines the relation between concepts and thereby constitutes their meaning^21^. Despite some variation in theoretical perspectives, these accounts converge on the assumption that abstract concepts require amodal or verbal distributional representations outside the experiential modal brain systems, as they transcend our senses. Although distributional language-based models do not specify the neuroanatomical basis of representations in the human brain, neuroimaging and theoretical work relates these distributional language-based representations to multimodal semantic hub regions such as anterior temporal cortex, middle temporal gyrus, inferior parietal cortex, or inferior frontal cortex^8,9,23–25^. These areas represent semantic information from different modalities.

Grounded cognition approaches provide a different perspective on the representation of abstract concepts and their neural implementation in the human brain. These theories propose that conceptual representations arise from distributed experiential modal representations encompassing processing of both external (perception) and internal sensations (proprioception, emotion, and introspection), as well as motor actions (e.g.^26–31^). Conceptual representations depend on activation patterns within brain areas dedicated to sensory, motor, introspective, and emotional processes.

Recent hybrid models of conceptual representations suggest a hierarchical organization of processing circuits, starting from lower-level modality-specific cortex, extending through bi-, tri-, or multimodal regions, and up to top-level amodal areas within heteromodal cortex^9,28,32–38^. This hierarchy is believed to reflect increasing levels of abstraction.

At first glance, due to the lack of a physical referent that can be perceived, it is difficult to conceive how abstract concepts can be represented in experiential modal brain systems. To specifically account for the representation of abstract concepts, refined grounded cognition theories propose a multiple representation view. This perspective encompasses abstract concept representations in various experiential modal brain areas, including those dedicated to sensory, motor, emotional, social, and mentalizing functions, as well as in language areas, depending on the specific semantic content of the concept^32,39–45^. Processing in multimodal and amodal areas complements these modal and linguistic representations to establish the meaning of abstract concepts^32^. This multiple representation view acknowledges that the content of abstract concepts is frequently related to emotional, mental and social constructs^40,46^. Furthermore, verbal communication or inner speech plays an important role in clarifying the frequently complex and heterogeneous meaning of abstract concepts^45,47^.

The relevance of visual, motor, emotional, mentalizing and social constellation features for some subgroups of abstract concepts has been shown in several neuroimaging and behavioral studies involving both healthy subjects and patients with brain lesions (for reviews, see^32,39^. This research indicated the involvement of the corresponding experiential modal brain areas in the processing of abstract concepts, with a high relevance of the respective feature type (e.g.^45,48–51^). Even abstract scientific concepts have been shown to depend on processing in experiential modal brain systems related to vision, motor action and social cognition^52,53^. Furthermore, behavioral, electrophysiological and neuroimaging studies demonstrated an increased experiential grounding of abstract concepts in experts compared to novices^53–56^. Such observations of expertise-related plasticity have been typically interpreted to reflect direct sensory or motor experience: Repeated interactions with situations, to which the abstract concept is applied, is assumed to establish memory traces in experiential modal brain circuits, which are re-activated when the concept is accessed^53^.

**Fig. 1:**
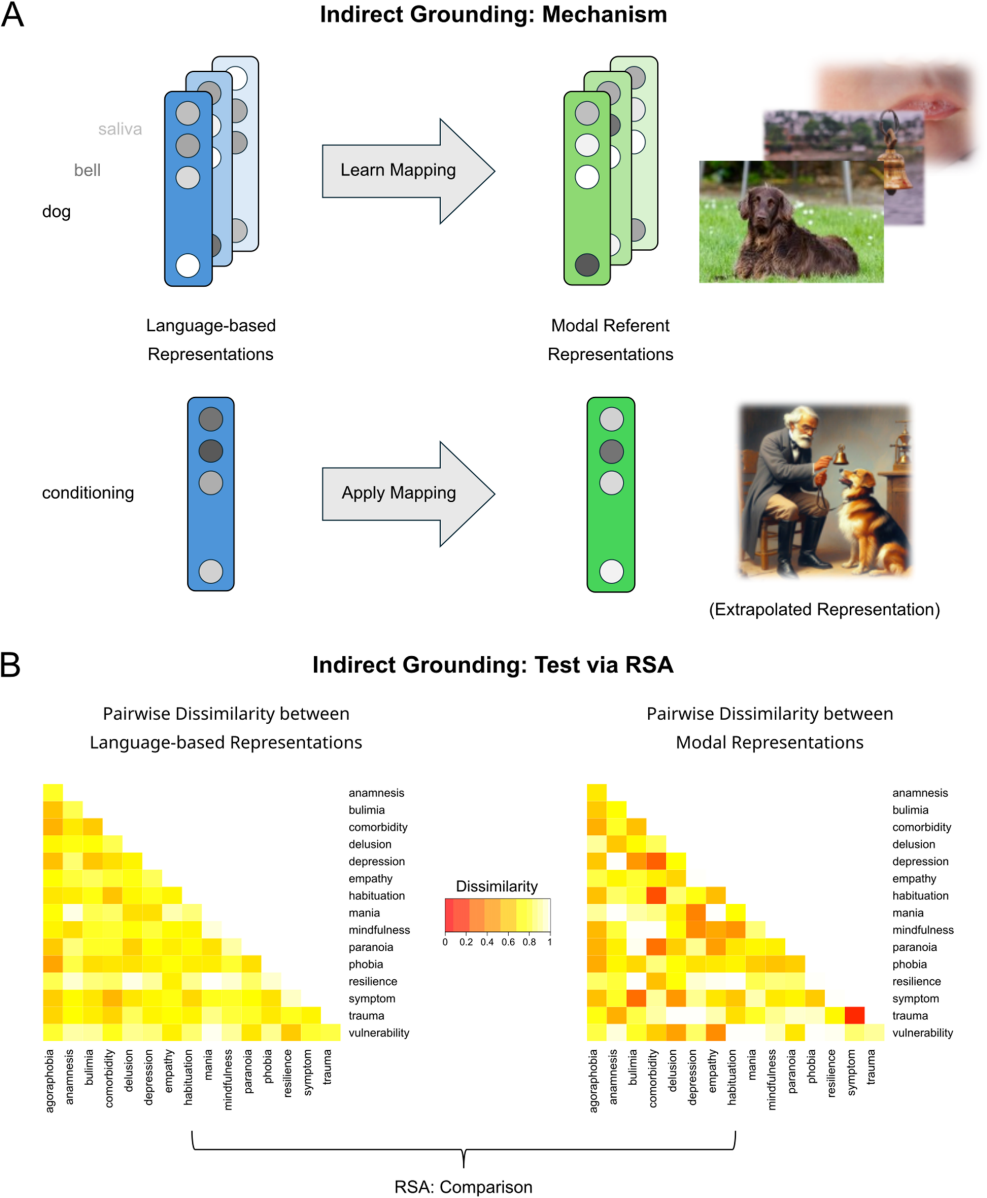
Indirect grounding, and its empirical test via representational similarity analysis (RSA) of functional magnetic resonance imaging (fMRI) data. **(A)** A mechanism for indirect grounding. We assume that speakers learn connections between linguistic and modal representations of their directly-experienced referents, for example via simultaneous exposure^63^. In line with Günther and colleagues^61^, this is realized as learning a mapping function between word meaning representations extracted purely from language data, and referents’ modal representations: Speakers learn to which extent each dimension/feature (depicted as greyscale circles) of the former corresponds to each dimension/feature of the latter. This mapping function can then be applied to other language-based word meaning representations to extrapolate modal representations for non-experienced referents. **(B)** An empirical test. If indirect grounding takes place, we should expect an isomorphism between language-based word meaning representations and modal representations. Representational similarity analysis allows us to detect such an isomorphism, by comparing (here: via their Spearman correlation) the dissimilarity matrix based upon distributed language representations (dissimilarity of semantic vectors from a word2vec Continuous Bag of Words (CBOW) model) with the dissimilarity matrix based upon neural activation patterns as measured with fMRI (dissimilarity of multi-voxel brain activity patterns in modal regions during word reading).

However, experiential grounding can also be achieved through an indirect mechanism that bypasses direct sensory or motor experience^57–59^. Such an indirect mechanism of experiential grounding might be particularly relevant for the acquisition of both concrete and abstract concepts, which are solely learned through verbal communication and reading text^26,60^. Recent theoretical and cognitive modelling work proposes the intriguing possibility of linking modal experiences with concepts for which direct experience is limited or absent^57,61^. This is achieved by extrapolating modal representations from available experiences relying solely on linguistic representations. In support of this idea, language representations based on word statistics have been shown to reflect real-world visual patterns^62^. This indirect grounding mechanism (see Fig. 1A) is conceptualized as a systematic mapping from distributional language-based representations onto modal representations, which is learned from concepts for which both linguistic and sensory experiences are available^61^. Crucially, once the mapping is learned, it can be applied to extrapolate sensory representations for words for which no such experience is available, based solely on their language-based representations.

In behavioral experiments utilizing computational models implementing this indirect grounding mechanism, human observers selected the model-predicted image above chance when instructed to choose the image that better represented the word’s meaning, as compared to an unrelated control image^61^. Similarly, in a training study, action congruency effects were observed for novel words learned from language alone^57^. While these findings suggest an indirect experiential grounding of concepts without direct experience, behavioral studies alone cannot confirm that these effects arise from extrapolated processing in experiential modal brain systems, a crucial assumption of grounded cognition theories of conceptual representations. It remains possible that language-based predictions of vision or action are exclusively computed within amodal or linguistic neural circuits located outside the experiential modal systems^64,65^. Thus, neural evidence is necessary to show that these effects arise from processing in modal brain systems, a crucial assumption of grounded cognition theories.

The proposal of indirect grounding through a mapping of language-based representations onto representations in modal brain systems can be elegantly tested through Representational Similarity Analysis (RSA) of functional magnetic resonance imaging (fMRI) data^66^. RSA (see Fig. 1B) allows to investigate the correspondence between the similarity relations of a set of words derived from a distributional language model and the similarity of multi-voxel fMRI activity patterns to these words^66^. Specifically, RSA allows for determining whether similarity based on a distributional semantic model as quantified in a representational dissimilarity matrix (RDM) can be captured in the activity patterns in modal brain areas, such as the visual or motor cortex. RSA of fMRI data has been previously applied to recover representational similarity based on visual features, semantic categories or text^67–69^ as well as based on experiential feature ratings or distributional language models^23,70–72^. In previous RSA studies on concrete concepts, similarity based on distributional language models was predominantly detected in multimodal hub areas such as the inferior frontal cortex^70,71^ and to a lesser extent in modal regions such as the motor cortex, challenging the idea of indirect grounding. However, the neural substrate of language-based semantic similarity of abstract scientific concepts has not been addressed. This concept category may provide a more appropriate test of the indirect grounding hypothesis due to the lack of a physical referent and the reduced involvement of direct experiential grounding.

To test the mapping of language-based representations of abstract scientific concepts onto representations in modal brain systems, an essential element of the indirect experiential grounding hypothesis, we used RSA to analyze the multi-voxel brain activation patterns in response to these concepts. All analyses were performed on a previously collected fMRI data set^53^. In this study, 64 words denoting abstract psychological concepts were presented within a lexical decision task (LDT) to undergraduate psychology students and to graduate psychologists during fMRI scanning. Visual, motor and emotional-social scene localizer tasks were run to identify experiential modal brain circuits involved in real visual perception, action, and emotional/social interaction. At present, there is no generally accepted operational definition of modality in terms of the optimal localizer tasks. In line with a recent definition of modality as “[a]ny discrete channel for transmitting, receiving, and/or representing information including but not limited to primary sensory data” (^73^, p. 258), we applied a pragmatic approach in designing the localizers and assessed brain activity induced by the presentation of stimuli from the corresponding channel or performing actions against a resting condition. As the modal content of abstract concepts is highly variable and not confined to narrow categories such as faces, houses, words or numbers^40^, our visual and emotional localizer tasks included stimuli from a broad range of categories of the corresponding modal channel. Although the operational definition of a modal brain region is a complex methodological issue, given the lack of a consensus in this respect, within a grounded cognition framework, the localizer tasks used in our study are appropriate to test the prediction that the distributional lexical semantics of abstract concepts map onto experiential brain regions involved in real perception and action.

Two model RDMs were formed to relate distributional language-based and experience-based semantic similarity to the fMRI activity pattern during scientific concept processing: The “language RDM” (Supplementary Figure S1A) was based on distributional language-based semantic representations as implemented in the computational model of indirect grounding^61^. The “experience RDM” (Supplementary Figure S1B) was based on the experiential properties of the presented abstract scientific concepts, collected from the participants of this study after the scanning session in a property listing task and coded according to modal feature types^40^. The experience RDM has a hybrid character, as generated properties related to a concept can be derived from both modal simulation based on direct experience as well as from retrieval of word associations^74^. Given this mixture of contributions from direct experience and word statistics, the RSA based on this experience RDM should be considered exploratory. If purely language-based distributional semantics support indirect grounding, the language RDM should allow for the recovery of semantic similarity in the modal cortex as defined by the localizer tasks, similarly to the experience RDM.

## Results

### Localizer tasks

**Fig. 2:**
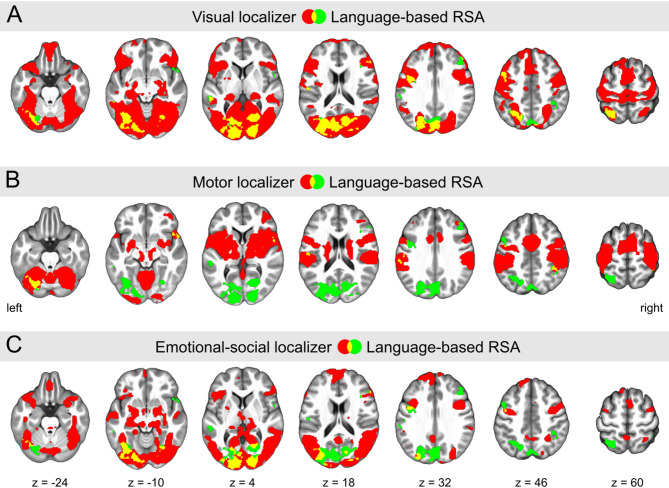
Results from Representational Similarity Analysis (RSA) for the language-based semantic space (shown in green), alongside the activation maps derived from the visual **(A)**, motor **(B)**, and emotional-social **(C)** localizer tasks (depicted in red). Areas where these maps overlap appear in yellow. 84.4% of significant voxels in the language-based RSA map overlap with those from the visual localizer, 9.0% with those from the motor localizer, and 41.1% with those from the emotional-social scene observation localizer. The RSA map was derived from t-testing similarity between the language Representational Dissimilarity Matrix (RDM) and the searchlights’ neural RDMs for significance across all 51 participants, irrespective of their expertise. All statistical parametric maps were thresholded at *p* < 0.001, family-wise error rate (FWE)-corrected (*p* < 0.05) at the cluster level, and overlaid onto the mean normalized skull-stripped T1 image using MRIcroGL^75^. Coordinates are in Montreal Neurological Institute (MNI) space. See also Supplementary Figure S3 for 3D renderings of these results.

When participants viewed images of objects as opposed to just looking at a fixation cross, significant increases in brain activation were detected bilaterally in the occipital and ventral temporal cortices, as well as in the posterior parietal cortex, parts of the precentral and postcentral gyri, the supplementary motor area, the lateral prefrontal cortex, the insula, in parts of the medial temporal lobe, and in the left putamen (Fig. 2A, depicted in red, and Supplementary Table S1).

Performing hand movements, relative to visual fixation, led to a pronounced increase in activation of the bilateral precentral and postcentral gyri, the supplementary motor area, lateral prefrontal areas, the basal ganglia, the thalamus, and extensive cerebellar regions, among others (Fig. 2B and Supplementary Table S2).

In the case of viewing emotional-social images versus neutral ones, significantly greater activation was observed bilaterally in the occipital and ventral temporal cortices, the middle and superior temporal gyri, the precentral gyrus, the ventrolateral and medial prefrontal cortex, the orbitofrontal cortex, and the medial temporal lobe, among other regions (Fig. 2C and Supplementary Table S3).

### RSA results

#### Language-based semantic representational similarity

A first set of RSA analyses examined the identification of semantic similarity of abstract psychological concepts based upon the distributional semantic language RDM from the multi-voxel MR activation patterns. A one-sample t-test for significant positive Spearman correlation between the language RDM and the whole-brain searchlights’ neural RDMs across the entire group of participants, irrespective of expertise, identified several brain regions illustrated in Supplementary Figures S2A and S5. The largest cluster, predominantly located in the occipital cortex, included parts of the cuneus, calcarine, lingual gyrus, and the inferior, middle, and superior occipital gyri. It also extended into the fusiform gyrus, the precuneus, and the superior parietal lobule. Other clusters encompassed parts of the left middle temporal gyrus, the left precentral and postcentral gyri, and the middle frontal gyrus bilaterally. The cluster in the postcentral gyrus had an overlap of 92.7% with the motor localizer. The precise locations and sizes of these clusters are detailed in Table 1. Moreover, Fig. 2 and Supplementary Figure S3 show the spatial overlap between these clusters and the activation maps derived from the three localizer tasks. Overall, 84.4% of the significant voxels in the language-based RSA map overlapped with those from the visual localizer task (Fig. 2A), while overlaps of 9.0% and 41.1% were observed with the motor (Fig. 2B) and emotional-social localizer (Fig. 2C) tasks, respectively. For a more detailed description of the cluster-wise percentages of overlap, please refer to the middle columns of Table 1. Nonparametric inference results based on permutation tests (see Supplementary Figure S6B) yielded a comparable pattern demonstrating the robustness of the findings.

A two-sample t-test did not reveal any significant differences in language-based representational similarity between graduate psychologists and undergraduate psychology students.

**Table 1:** Brain regions revealed by a one-sample t-test for significant positive Spearman correlation between the language Representational Dissimilarity Matrix (RDM) and the searchlights’ neural RDMs across all participants (*n* = 51). A voxel-height threshold of *p* < 0.001 and family-wise error rate (FWE) correction (*p* < 0.05) at the cluster level were applied. Coordinates are in Montreal Neurological Institute (MNI) space. The “Overlap with localizer maps” columns indicate the percentage of voxels within each cluster that coincide with activations identified by the visual, motor, and emotional-social localizer tasks. L: left; R: right.

Brain region	Cluster size (in voxels)	Overlap with localizer maps			Peak voxel			
		visual	motor	emotion	x	y	z	z-score
Lingual gyrus (R)	8953	91.1%	5.1%	45.8%	20	-86	0	5.56
Superior occipital gyrus (L)					-10	-96	4	5.55
Calcarine (R)					16	-94	6	5.44
Lingual gyrus (R)					24	-66	-2	5.25
Cuneus					0	-76	36	5.07
Middle occipital gyrus (L)					-32	-88	22	5.00
Fusiform gyrus (L)					-34	-66	-14	4.72
Superior parietal lobule (L)					-32	-64	56	4.43
Superior parietal lobule (L)					-26	-62	50	4.33
Calcarine (R)					16	-70	12	4.29
Calcarine (L)					-18	-72	6	4.27
Cuneus (L)					-6	-90	24	4.22
Cuneus (R)					2	-84	22	4.21
Calcarine (L)					-4	-80	14	4.13
Superior occipital gyrus (L)					-26	-82	30	4.10
Inferior occipital gyrus (L)					-36	-74	-10	4.01
Cuneus (L)					-12	-74	26	3.96
Precuneus (L)					-4	-78	48	3.94
Cerebellum (L)					-18	-68	-22	3.92
Fusiform gyrus (R)					30	-64	-12	3.72
Middle occipital gyrus (L)					-36	-90	6	3.66
Precuneus (R)					8	-64	36	3.63
Middle occipital gyrus (R)					32	-86	16	3.60
Superior parietal lobule (L)					-32	-56	66	3.51
Middle occipital gyrus (L)					-26	-98	12	3.50
Lingual gyrus (L)					-4	-76	2	3.48
Middle occipital gyrus (R)					34	-70	26	3.48
Inferior parietal lobule (L)					-30	-74	44	3.28
Postcentral gyrus (L)	191	24.6%	92.7%	0.0%	-56	-20	24	4.61
Supramarginal gyrus (L)					-64	-28	34	3.82
Precentral gyrus (L)	465	89.9%	16.3%	34.6%	-44	-2	30	4.41
Middle frontal gyrus (L)					-48	10	44	3.81
Precentral gyrus (L)					-44	-4	42	3.65
Middle temporal gyrus (L)	199	35.2%	2.5%	0.0%	-56	-34	6	4.27
Middle temporal gyrus (L)					-58	-40	-6	3.93
Inferior parietal lobule (R)	212	0.0%	42.0%	0.0%	40	-42	44	4.09
Inferior parietal lobule (R)					44	-48	50	3.59
Inferior parietal lobule (R)					38	-52	44	3.46
Middle frontal gyrus (R)	271	30.3%	2.6%	8.9%	44	28	32	3.88
Middle frontal gyrus (R)					46	38	34	3.50
Inferior frontal gyrus, triangular part (R)					50	30	24	3.39
Rolandic operculum (R)	157	29.3%	78.3%	7.6%	54	4	8	3.65
Superior temporal pole (R)					56	14	-10	3.44

### Experience-based semantic representational similarity

**Fig. 3:**
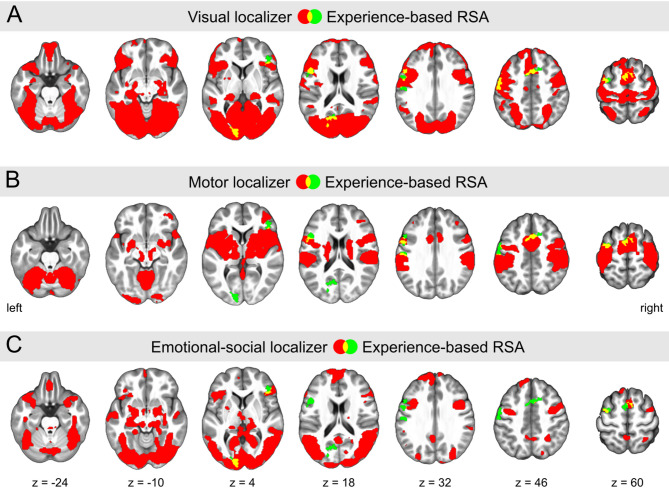
RSA results for the experience-based semantic space (in green), with the addition of the activation maps from the visual **(A)**, motor **(B)**, and emotional-social **(C)** localizer tasks (in red). Overlapping regions are highlighted in yellow. The RSA map was derived from a one-sample t-test (*n* = 51). All statistical parametric maps were thresholded at *p* < 0.001 (cluster-FWE-corrected, *p* < 0.05). Significant voxels of the experience-based RSA map aligned to 66.9% with the visual localizer, 47.8% with the motor localizer, and 21.4% with the emotional-social localizer. Coordinates refer to MNI space. See also Supplementary Figure S4 for 3D renderings of these results.

A second set of RSA analyses focused on recovery of semantic similarity of the abstract scientific concepts based upon participants’ property listings from MR activation patterns. Across all participants, we found significant Spearman correlations between the experience RDM and the searchlights’ neural RDMs in the left occipital cortex, left precentral and postcentral gyri, left middle frontal gyrus, bilateral inferior frontal gyri, supplementary motor area, and the middle cingulate cortex. The results are presented in Supplementary Figures S2B, S5, and Table 2. Nonparametric inference results based on permutation tests (Supplementary Figure S6D) revealed a quite comparable pattern. Overlap with the localizer activation maps is shown in Fig. 3 and Supplementary Figure S4. Specifically, 66.9% of significant voxels within the experience-based RSA map coincided with the visual localizer’s activation map (Fig. 3A). Overlap with the motor (Fig. 3B) and emotional-social localizer (Fig. 3C) tasks was observed at 47.8% and 21.4%, respectively. Detailed breakdowns of these overlaps, categorized by cluster, are further delineated in Table 2. Representational similarity between the experience RDM and the searchlights’ neural RDMs was not significantly different between graduate psychologists and psychology students.

**Table 2:** RSA results from a one-sample t-test assessing significant representational similarity between the experience RDM and the searchlights’ neural RDMs (*n* = 51). The statistical parametric map was thresholded at *p* < 0.001 (voxel-level), FWE-corrected (*p* < 0.05) at the cluster level. Coordinates refer to MNI space. For each cluster, the overlap with localizer maps is reported as the percentage of voxels that were also significantly activated during the visual, motor, and emotional-social localizer tasks. L: left; R: right.

Brain region	Cluster size (in voxels)	Overlap with localizer maps			Peak voxel			
		visual	motor	emotion	x	y	z	z-score
Superior occipital gyrus (L)	201	100.0%	0.0%	68.7%	-14	-94	4	4.38
Superior occipital gyrus (L)					-18	-84	10	3.63
Middle occipital gyrus (L)					-20	-82	18	3.51
Inferior frontal gyrus, opercular part (L)	758	67.2%	47.4%	0.5%	-48	16	18	4.31
Postcentral gyrus (L)					-54	-18	26	4.13
Precentral gyrus (L)					-56	-2	42	3.78
Precentral gyrus (L)					-56	4	32	3.75
Precentral gyrus (L)					-56	2	44	3.71
Middle frontal gyrus (L)					-50	12	40	3.68
Postcentral gyrus (L)					-56	-14	46	3.53
Supplementary motor area (L)	778	68.4%	80.7%	31.7%	-8	-4	68	4.15
Precentral gyrus (L)					-38	0	64	4.08
Supplementary motor area (R)					6	14	52	3.89
Supplementary motor area (L)					-10	-8	70	3.78
Supplementary motor area (L)					-2	14	54	3.77
Supplementary motor area (R)					6	8	54	3.66
Middle cingulate cortex (L)					-6	14	36	3.65
Inferior frontal gyrus, orbital part (R)	194	17.5%	23.7%	25.8%	38	42	-4	4.07
Inferior frontal gyrus, triangular part (R)					48	36	4	3.86
Inferior frontal gyrus, triangular part (R)					44	28	0	3.79
Calcarine (L)	228	73.7%	0.0%	10.1%	-8	-64	22	3.71
Calcarine (L)					-10	-64	14	3.70

### Comparison of language- and experience-based semantic representational similarity

The language and the experience RDMs were slightly, but significantly, correlated (Spearman’s rho = 0.08, *p* = 0.002). In line with the putative hybrid processes underlying property generation, this correlation indicates that part of the semantic similarity captured by the property listings is rooted in word statistics. In two exploratory analyses, we compared language- and experience-based semantic representational similarity to reveal possible commonalities and differences. First, we identified in a conjunction analysis brain regions allowing for detection of both language- and experience-based semantic similarity. Second, we tested for brain regions allowing for a more efficient recovery of semantic similarity based on the language RDM than that based on the experience RDM and vice versa. Both analyses, performed across all 51 participants using a paired t-test, were implemented in Statistical Parametric Mapping (SPM12). The conjunction of language- and experience-based similarities was significant in the left lingual gyrus, superior occipital gyrus, and calcarine, as well as in the left inferior and middle frontal gyri (Supplementary Figure S2C and Supplementary Table S4). There were no significant differences between language- and experience-based semantic similarities.

## Discussion

The present study assessed the possibility of a mapping of distributional language-based representations onto experiential modal representations. Specifically, we used RSA of MR activation patterns to test the prediction derived from the indirect grounding hypothesis^57–59,61^ that the similarity of language-based semantic representations as determined in the language RDM can be identified in activity patterns in modal brain regions. In line with the indirect grounding hypothesis, the similarity of abstract scientific concepts based on distributed semantic language representations was reflected in MR activity patterns in the occipital cortex as well as in fronto-parietal motor regions (pre- and postcentral gyri, and middle frontal gyrus), which were also activated by real visual perception and motor action. Similarity of abstract scientific concepts based on experiential features derived from the property listing task could also be captured in occipital visual areas and in fronto-parietal motor regions (pre- and postcentral gyri, supplementary motor area, and inferior frontal gyrus). The role of occipital areas in visual representations has been highlighted by earlier RSA studies demonstrating their sensitivity to visual object similarity^76^. Correspondingly, action similarity could be recovered from MR activity patterns in the postcentral gyrus in previous RSA work^77^, thereby indexing its role in action representations. Consistent with the present results, language-based similarity between action verbs could be identified in frontal motor areas^70^.

In contrast to the present findings on abstract concepts, previous RSA studies on concrete concepts recovered similarity based on distributional language models predominantly in multimodal hub areas such as the inferior frontal cortex^70,71^ and to a lesser extent in modal regions such as the motor cortex, challenging the idea of indirect grounding. However, as already mentioned in the introductory section, abstract concepts may provide a more appropriate test of the indirect grounding hypothesis due to the lack of a physical referent and the reduced involvement of direct experiential grounding. The meaning of abstract concepts, such as scientific psychological concepts, must therefore be elaborated to a large extent through language^42^, thereby enhancing the mapping of distributed language representations onto representations in modal brain systems. In contrast, the meaning of concrete concepts is predominantly established through direct sensory or motor experiences of the referent^74^, and to a lesser extent through language, thereby reducing the contribution of indirect grounding. As the present study included only abstract concepts, a possible differential contribution of indirect grounding mechanisms to concrete and abstract concepts will need to be elucidated in future studies.

Nevertheless, in advancing earlier work, the present study shows for the first time that visual and motor brain areas encode language-based similarity of abstract scientific words and not only similarity of physical experiential features or that of concrete concepts. In support of the proposed indirect grounding hypothesis, the present observation provides evidence for a mapping of distributed language representations onto memory traces in modal brain systems, even for highly abstract psychological concepts. Identification of semantic similarity did not significantly differ between psychology students and graduates, suggesting that this mapping was not affected by the level of academic expertise. Interestingly, an earlier RSA study^78^ on semantic processing of concrete words recovered semantic similarity based on word2vec vectors of distributed language representations from an ROI in visual cortex identified as the visual word form area based on functional and anatomical criteria. Our study extends this work by showing that distributional language representations are mapped on activity patterns in various parts of the visual system, also activated by non-verbal visual stimulation.

In addition to modal brain regions as defined by the localizer tasks, language- and experience-based similarity was reflected in multimodal semantic hubs^8,9,24,25,38,79^. These areas represent semantic information from different modalities. Consistent with previous RSA studies (e.g.^70–72,80^) and with the function of these areas as higher-level semantic convergence zones, the temporal pole, middle temporal gyrus, supramarginal gyrus, precuneus, inferior frontal gyrus, and middle frontal gyrus were sensitive to language-based semantic similarity in the present study. Some of these hub regions, the inferior and middle frontal gyri, were also sensitive to experience-based semantic similarity. However, a direct comparison of language-based vs. experience-based similarities did not reveal significant differences. This indicates that both types of semantic similarities were comparably recovered from modal and multimodal brain areas.

Most likely, experience and language RDMs similarly capture conceptual representations formed by indirect grounding mechanisms: The language RDM only reflects statistical word co-occurrence as determined by the Continuous Bag of Words (CBOW) algorithm^61^ – and without access to any direct sensory information, only indirect experiential grounding can possibly be captured. The experience RDM reflects direct modal experience with the referent, but most likely also a contribution from statistical word co-occurrence: Generated properties related to a concept might be derived from both modal simulation based on direct experience as well as from retrieval of word associations^74^. In line with these assumptions regarding the mechanisms underlying property generation, the language and the experience RDMs were slightly, but significantly, correlated. The contribution of language-based similarity to both RDMs may explain why they allow identification of semantic similarity in a quite comparable set of brain areas. Given its hybrid character, our experience RDM cannot be compared with the pure experience RDM in earlier work^72^, which is based on word ratings of direct experience.

We would like to emphasize at this place that the present study tested the mapping of language-based representations of abstract scientific concepts onto representations in modal brain systems, an essential element of the indirect experiential grounding hypothesis. The process of extrapolation itself is beyond the scope of the present work. The process of extrapolation and the nature of the extrapolated representations themselves can only be investigated within a training study using novel words such as in the work by Günther and colleagues^57^. Such a training study must include novel words, for which direct sensory experience is absent, as well as novel words, for which sensory experience is available. For novel words without sensory experience, sensory extrapolation, if there is any, can occur via linguistic associations with words, for which both linguistic and sensory experiences are available and for which a systematic mapping from distributional language-based representations onto modal representations had already been established. Nevertheless, the mapping from distributional language-based representations onto modal representations is an essential element of the indirect grounding hypothesis, which was tested in the present study at a neural level.

Furthermore, brain activity measures, such as fMRI, provide only correlative evidence, but do not prove the causal relevance of the modal neural circuits involved in the indirect grounding of abstract scientific concepts. Future transcranial magnetic stimulation or studies in brain-lesioned patients are needed to determine whether these experiential brain circuits associated with indirect grounding are functionally relevant for conceptual processing. It also remains to be demonstrated whether indirect grounding of conceptual knowledge generalizes to other types of abstract concepts or to concrete concepts.

In conclusion, the present study provides the first neural evidence for the mapping of distributional language representations of abstract scientific concepts onto experiential modal representations. Specifically, similarity of abstract psychological concepts derived from distributed language-based representations could be recovered from the multi-voxel activity pattern within occipital and fronto-parietal cortex, overlapping with activation induced by real visual perception and hand action, and not exclusively from multimodal hub regions. The present findings therefore indicate that patterns of statistical word co-occurrence, as captured by distributional language models, map onto modal visual and motor representations. This mapping between language and the visuo-motor system documented in the present study is a fundamental element of the proposed indirect conceptual grounding mechanism. This indirect grounding mechanism provides sensory-motor experience for the unseen and enriches conceptual knowledge for concepts learned exclusively from language.

## Methods

### Participants

A first group of participants consisted of 25 graduate psychologists (21 females; mean age: 28.4 years; standard deviation, SD: 2.7 years), all of whom had attained at least a Master’s degree. These participants were either in the process of training as psychological psychotherapists or had already completed their psychological psychotherapy education. Additionally, 28 undergraduate psychology students from Ulm University (20 females; mean age: 23.1 years; SD: 3.0 years) participated in the study. In our predecessor article^48^, two participants had to be excluded due to insufficient feature listings in the property listing task, which would have been problematic for data modeling. However, this is not an issue for the RSA analyses described in the current article, which is why these aforementioned two participants have been included in the present work. However, due to an erroneous calculation of required MR volumes, the MR acquisition for two female undergraduate psychology students did not sufficiently cover the very last trial, preventing estimation of the neural response for the associated abstract concept. Therefore, the data from these two participants were excluded from further analysis. After this exclusion, the group of undergraduate students (*n* = 26) had a mean age of 22.9 years (SD: 3.0 years). All 51 individuals were healthy German speakers, and except for one individual in each group, all were right-handed. The research adhered to the ethical guidelines outlined in the Declaration of Helsinki, though it was not pre-registered. The study and its experimental procedures were approved by the ethics committee of Ulm University. Before commencing the experiment, participants provided their written informed consent and they were offered either financial remuneration or course credits in exchange for their participation.

### Lexical decision task

In the LDT, we employed 64 nouns denoting abstract psychological scientific concepts (e.g., “flashback”, “melancholy”) that were sourced from a pilot study detailed in Ulrich et al.^53^. Each noun was required to satisfy specific criteria. Firstly, all participants of the pilot study needed to be fully acquainted with the noun. Secondly, graduate psychologists had to report a more concrete experience with the noun compared to undergraduate psychology students, with a minimum difference in ratings of 0.4 (mean difference: 1.3, SD: 0.7). Thirdly, the ratings for “concrete experience” by graduate psychologists needed to be no less than 3.0. When testing the full set of nouns for group differences, the disparities in fluency and concrete experience were both significant as planned: “fluency” for graduate psychologists was 5.1 ± 0.4, compared to undergraduate students at 4.1 ± 0.7, t(126) = 10.20, *p* < 0.001; “experience” for graduate psychologists stood at 4.7 ± 0.7, against undergraduate students at 3.4 ± 0.7, t(126) = 10.36, *p* < 0.001. From the pool of remaining abstract scientific nouns not utilized, another set of 64 words was selected to construct pronounceable yet meaningless pseudowords. This involved altering one vowel and one consonant in the original noun with a different vowel and consonant. Real words and pseudowords were matched based on their length (mean number of letters in words = 12.0 (SD = 4.8), mean number of letters in pseudowords = 12.0 (SD = 2.8); t(126) = 0.02, *p* = 0.982). A comprehensive list of stimuli is available in Supplementary Table S5. Additionally, for two practice sessions, 10 words and 10 pseudowords not featured in the main study were prepared.

Stimuli were displayed using a white typeface against a black backdrop, centered on a 32” LCD screen (NordicNeuroLab AS, Bergen, Norway), with a resolution of 800 × 600 pixels and a refresh rate of 75 Hz. This setup was positioned to project through the bore of the MRI scanner and reflected towards the participant via a mirror attached to the MRI head coil. Participants had to quickly and accurately determine if targets were real words or pseudowords by pressing a button for pseudowords (go trial), but refraining from responding to real words (no-go trial). This go/no-go response mode ensured that potential activity within the motor system to abstract concepts was not compromised by an overt motor response. Each trial spanned 2400 ms, structured as follows: Initially, a fixation cross appeared for 500 ms, succeeded by the presentation of the target (either a word or pseudoword) for 400 ms, and then a blank screen for 1500 ms. Following each trial, the screen would go blank for a variable intertrial interval, averaging 3173 ms (standard deviation: 3435 ms), before the commencement of the next trial. The task was practiced on two occasions: initially outside the MR scanner with 10 words and 10 pseudowords during the instruction phase, and then again inside the MR scanner, just before the LDT experiment began, with five words and five pseudowords. After the scanning session, participants rated all abstract concepts from the LDT, along with 16 concrete concepts (as reference), on a 6-point Likert scale indicating how abstract or concrete each concept was for them (1 - very abstract, 6 - very concrete). The 58 abstract psychological concepts included in the RSA (for the reasons to exclude 6 out of 64 words from the initial word set, see below) received a mean abstractness rating of 2.74 (SD = 0.58), while the 16 concrete control concepts had an average rating of 5.99 (SD = 0.06). Hence, all abstract concepts used in the present study can be considered as “abstract”, in the sense that they are distributed on the lower end of the abstractness-concreteness continuum.

A preliminary sequence of trials was developed using the program ‘optseq2’ (http://surfer.nmr.mgh.harvard.edu/optseq/; see also^81^. The sequence of trials and their onsets, as outputted by the software, were adjusted to ensure that a particular condition did not recur more than three consecutive times. Additionally, the onset times were jittered by the random addition of fractions of the fMRI repetition time. While this trial sequence was held constant across all participants, for each participant and condition, the stimuli were allocated to their designated trial positions on a random basis. As a result, although each participant encountered the same set of 64 abstract concepts and 64 pseudowords, the order in which these stimuli appeared was unique to each individual, with each stimulus presented only once within a single experimental run. Given that word order was fully randomized across participants, any potential inhibition of brain activation^82^ for words immediately following go trials (pseudowords) would have affected individual words in a non-systematic manner, making it unlikely for such effects to confound RSA results.

The software employed for presenting the stimuli (and capturing behavioral responses) was Presentation 18.1 (Neurobehavioral Systems Inc., San Francisco, USA).

### Localizer tasks

After the lexical decision task, participants underwent three localizer tasks designed to identify brain areas involved in visual, motor, and emotional-social processing. The protocols for the visual and motor tasks were based on previous research (e.g.^49^). Stimuli were displayed on the previously mentioned LCD screen against a black background.

For the visual localizer task, there were 120 images (dimensions: 440 × 440 pixels) depicting both animate and inanimate entities, equally divided. These images were shown across six task blocks, separated by periods of rest. Each task block had a duration of 24 s, within which 20 images were shown. Each image appeared for 500 ms and was followed by a white fixation cross that varied in duration between 500 and 900 ms. The fixation cross was also present during the rest periods, which lasted 27.8 s. Participants were instructed to pay close attention to every stimulus presented.

During the motor localizer task, participants were asked to respond to visual cues by vigorously squeezing a flexible hand exercise ball with either their left or right hand. The cues consisted of white arrows pointing left (<< ) or right (>> ), indicating which hand should be used. A cue appeared on the screen for 300 ms, followed by a black screen to mark the interval until the next cue. These intervals between cues were randomized, ranging from 1.28 to 2.78 s. The task was structured into eight blocks, alternating between the left and right hands, with each block dedicated to one hand at a time. Four blocks were designated for the left hand and four for the right hand, and during each block, participants were required to compress the ball a total of ten times, using only the specified hand. Task blocks were interspersed with rest blocks, with all task and rest blocks lasting 26 s each. During the rest periods, participants needed to focus on a white cross that appeared and disappeared in a manner similar to the arrow cues during the task blocks. The concluding 4 s of all task and rest blocks featured a black screen. Overall, participants were instructed to restrict movement solely to the hands.

The emotional-social scene observation localizer task incorporated 80 images featuring neutral (40), pleasant (20), or unpleasant (20) visual scenes, sourced from the International Affective Picture System^83^ (IAPS; stimulus indices are provided in Supplementary Table S6). The images of pleasant and unpleasant scenes depicted either humans or animals in emotionally charged situations, either in solitude or interacting with others. Neutral images portrayed individuals with an impartial facial expression, alongside various animate (e.g., a mushroom) and inanimate objects (e.g., a spoon). Pleasant images included scenes of animals generally regarded as adorable (e.g., a kitten), infant humans, or individuals relishing moments of life (e.g., happy grandfather embracing his grandchildren). Unpleasant images consisted of scenes such as suffering animals, malnourished children, or a car accident. The selection ensured a significant difference in valence between pleasant (mean: 7.9, range: 7.5–8.2) and unpleasant (mean: 2.2, range: 1.8–2.8; t(38) = 57.73, *p* < 0.001) images, without a notable difference in arousal levels (pleasant: mean: 5.3, range: 4.3-7.0; unpleasant: mean: 5.5, range: 4.0-6.9; t(38) = -0.52, *p* = 0.609). Valence ratings for neutral scenes were significantly distinct, being lower or higher than those for pleasant or unpleasant scenes, respectively (mean: 5.0, range: 4.9–5.2; neutral vs. pleasant: t(58) = -67.48, *p* < 0.001; neutral vs. unpleasant: t(58) = 45.75, *p* < 0.001). The arousal ratings for neutral scenes were significantly lower on average compared to the other two categories (mean: 3.1, range: 1.8-6.0; t(78) = 12.89, *p* < 0.001). The 40 neutral images were split into two subsets of 20 each. Images had dimensions of 512 × 384 pixels. The presentation of the (randomly ordered) stimuli was organized into blocks, each lasting 23.8 s. Similar to the visual localizer task, within this duration, 20 images alternated with a fixation cross (image duration: 500 ms, fixation cross: 500–900 ms), with each block featuring only one type of stimulus category. There were three blocks each for pleasant and unpleasant stimuli, and six for neutral stimuli, allowing each image to be displayed three times. The sequence of (un)pleasant and neutral blocks was interspersed, with the precise sequence of pleasant versus unpleasant blocks being randomized. A fixation cross appeared for 5.8 s between blocks. Participants were instructed to closely attend to all presented stimuli.

### Property listing task

Following MR scanning, participants completed a property listing task aimed at capturing the individual meanings tied to each abstract psychological concept encountered during the LDT. The property listing task closely mirrored the approach detailed in prior research (for further details, see^40^). Participants were given a questionnaire that included all of the real-word stimuli from the LDT. They were prompted to spontaneously list three to four properties, situations or associations triggered by the specific concept, with an emphasis on avoiding mere synonyms. To ensure clarity, the instructions were illustrated through two example words not included in the LDT. The questionnaire came in four variants, each presenting the words in a unique randomized sequence. These versions were distributed among participants in a counterbalanced manner.

The properties produced were categorized by author A.B., who was unaware of the participants’ group affiliation, into one or several of the following feature categories: “visual”, “motor-related”, “acoustic”, “tactile”, “olfactory”, “gustatory”, “interoceptive”, “social constellation”, “mental state”, “emotion”, and “verbal association”. It is important to note that the identification of a verbal association prevented multiple categorizations. While the first seven sensorimotor-related categories are quite straightforward, the “social constellation” category encompassed features and situations that involve the presence of or interaction among two or more individuals. The category “mental state” was used for features and situations associated with mental processes (e.g., “expectation”). The “emotion” category subsumed features/situations indicative of affective evaluation. The “verbal association” category was reserved for properties/situations that, rather than describing the actual concept, were thematically or symbolically connected to it. For each word and participant, the proportion of produced properties per feature category was calculated. Six participants did not generate properties for all words (1–3 words per participant were missing; in total, properties for 9 words were missing). Rather than substituting the missing data when calculating the experience RDM (see below), we averaged the frequency of generated properties across available participants for each concept.

To assess the consistency of classifications between raters, the properties were independently evaluated by another author (N.M.T.), who also had no knowledge of the participants’ group memberships. We employed intra-class correlations^84^ (ICC), utilizing a two-way model of the agreement of both raters^85^. The ICC values varied from 0.30 to 0.82, with the lowest value of 0.30 attributed to the “olfactory” category, which constituted merely 0.2% of the total classifications. To adjust for classification frequency, we calculated a weighted average of the ICC scores of all categories, i.e., each category was weighted by its frequency (mean frequency of both raters) and the mean of these weighted scores was calculated. This approach yielded a weighted ICC mean of 0.67, indicative of moderate inter-rater reliability^86^.

### MRI data acquisition

Magnetic resonance imaging was conducted using a 3 Tesla MAGNETOM Prisma equipped with a 64 channel head/neck coil (Siemens AG, Erlangen, Germany). Functional images representing the T2*-weighted blood oxygenation level-dependent (BOLD) signal were acquired employing an echo-planar (EPI) pulse sequence. The parameters for this sequence included a repetition time (TR) of 2000 ms, echo time (TE) of 34 ms, a flip angle of 90°, bandwidth of 2268 Hz/Px, a PAT factor of 2 (GRAPPA mode), a field of view (FOV) of 192 mm, and a matrix size of 76 × 76. Slice acquisition was ascending and yielded 33 transversal slices, with slice thickness being 3.5 mm, and having an interslice gap of 0.88 mm, resulting in a voxel size of 2.53 mm × 2.53 mm × 4.38 mm. The number of EPI volumes collected varied across tasks, with the LDT requiring 360 volumes (12.1 min), the visual localizer task 177 volumes (6.0 min), the motor localizer task 230 volumes (7.8 min), and the emotional-social scene observation localizer task 184 volumes (6.3 min).

Subsequently, a high-resolution T1-weighted structural image was obtained using a magnetization-prepared rapid gradient echo (MPRAGE) sequence. The parameters for this sequence were a TR of 2300 ms, a TE of 2.32 ms, an inversion time of 900 ms, a flip angle of 8°, a bandwidth of 200 Hz/Px, a PAT factor of 2 (GRAPPA mode), a FOV of 240 mm, and a matrix size of 256 × 256, leading to a voxel size of 0.90 mm × 0.94 mm × 0.94 mm. The slices were oriented sagittally. Scan time was approximately 5.4 min.

### MR image preprocessing, activation analysis, and representational similarity analyses

The preprocessing and statistical analyses of the imaging data, except for RSA, were performed using SPM12 r6225 (Wellcome Department of Cognitive Neurology, London, UK) running on MATLAB R2021a (MathWorks Inc., Natick, MA, USA). For the purposes of skull-stripping and image normalization to MNI space, the Computational Anatomy Toolbox^87^ 12 (CAT12.8.2, version 2170) was employed. RSA was conducted using the “rsatoolbox"^88^. While implementations for both MATLAB and Python are available, in the current study, the (newer) Python implementation (https://rsatoolbox.readthedocs.io) was utilized, specifically version 0.1.3, under Python 3.11.4. A list of Python libraries relevant to this paper, along with their versions and dependencies as determined by “pipdeptree” (version 2.15.1), is available in the Supplemental information.

First, for each participant, the initial four EPI volumes of each task-based series were removed. A slice time correction, with the reference slice set to 17, was applied exclusively to the EPI images of the lexical decision task. Following this, the EPI images of each series were spatially realigned to their own series-specific mean image. The mean EPIs from the localizer tasks then served to coregister all localizer EPIs to the mean EPI from the LDT. Subsequently, a skull-stripped, bias-, noise- and global intensity-corrected version of the T1 image in native space was created. This was achieved by running the segmentation module of the CAT12 toolbox in “Expert Mode”, mostly with default settings, except for enabling the option to write a bias-, noise- and global intensity-corrected T1 image in native space. Using SPM12’s imcalc routine, this corrected T1 image underwent skull-stripping by filtering it with the p0 label image (which was also a product of the segmentation step), where the p0’s voxel values were greater than zero. Finally, this skull-stripped T1 image was coregistered to the LDT’s mean EPI, and the transformation parameters were also applied to the original T1 image.

Now that the functional and anatomical images were aligned while still in native space, the LDT data were modeled at the first level to obtain an estimate for each word trial’s activation. We employed the “Least Squares-Single” (LSS) approach^89,90^, which uses separate general linear models (GLM) to estimate the activation of each trial, with one regressor representing only a single real word trial of interest, and two additional regressors: one for all other real word trials and another for all pseudoword trials. Resulting stick-functions were convolved with the canonical hemodynamic response function (HRF). The parameters from spatial realignment were incorporated into the design matrix as additional regressors. To mitigate the impact of low-frequency scanner drifts, the data underwent high-pass filtering (cutoff: 128 s). Temporally correlated residual errors were addressed using a first-order autoregressive model (AR1). The individual skull-stripped T1 image served as an explicit mask, with the implicit masking threshold disabled. Following estimation of each model, a contrast image was generated to represent activation estimated for the single word trial at hand. The contrast images, after being renamed according to the semantic concept they represented, constituted the inputs for the RSA toolbox. However, before commencing the actual analysis, as a further preparatory step, two types of model RDMs were constructed to characterize the semantic relationships between the abstract concepts from the LDT by two different measures of semantic similarity. The first RDM was generated from semantic vectors based on a distributional semantic language model. Specifically, we employed the “de_wiki” space, available at https://sites.google.com/site/fritzgntr/software-resources/semantic_spaces (last accessed July 25, 2023; see also^91^). This semantic space was created using the CBOW variant of the word2vec model^92^, trained on a 2017 dump of the German Wikipedia, encompassing 1.5 billion words. From this language model, we extracted the specific 400-dimensional feature vectors for the alphabetically ordered concepts employed in the LDT, resulting in a 58 × 400 matrix. The matrix included only 58 concepts (instead of 64) because the features of the following six concepts were not found verbatim in the database (which only includes single words), leading to their omission from all further analyses: “behavioral experiment”, “catastrophization”, “delayed reward”, “imagination exercise”, “personality accentuation”, and “safety behavior”. Conversion of the feature matrix to an RDM was achieved by calculating the pairwise correlation distance (1 - Pearson’s correlation coefficient) across the set of concepts. This “language RDM” is shown in Supplementary Figure S1A. In a corresponding procedure, a second RDM, the “experience RDM” (see also^71,72^), was created on the basis of the experiential properties that participants had listed for those 58 concepts during the property listing task. These properties were classified as related to modal features (visual, motor, etc.) according to a coding scheme described in Harpaintner et al.^40^. The frequencies of generated properties were averaged across participants, yielding a 58 × 11 matrix that was again converted into a corresponding RDM, a visualization of which is presented in Supplementary Figure S1B. Both RDMs were saved as “hdf5” files for easier reuse.

Next, for each participant, the contrast images representing activation associated with each concept were loaded using the Python package “nibabel” (version 5.1.0). The images were arranged alphabetically, consistent with the order used for creating the model RDMs. The voxel data from these images were organized into a four-dimensional NumPy array. Then, across the whole brain, searchlight centers and neighbors were calculated, applying a masking threshold that required at least 50% of voxels to be within each searchlight sphere of radius 3 voxels, a setting relevant at brain boundaries. This step enabled the computation of a “neural” RDM for each searchlight by calculating the correlation distance between each pair of concepts’ normalized multi-voxel activation pattern. Finally, the similarity between each searchlight’s neural RDM and the language RDM, and between the neural RDMs and the experience RDM, was assessed by calculating Spearman’s rank correlations. These coefficients were saved as NIfTI images using “nilearn” (version 0.10.1). In order to normalize these images to MNI space, the CAT12 toolbox was employed for a second time, using the already coregistered, but still skull-containing T1 image as input. Importantly, the toolbox was configured to output the forward deformation field, as well as the bias-, noise- and global intensity-corrected T1 image in normalized space (for creating a normalized group mean T1 image used for visualizing the results). The voxel size for normalized images was set to 1 mm. The resulting deformation field was then applied to the images containing the RSA Spearman correlation coefficients, thus achieving normalization to MNI space. After this step, voxels had a size of 2 mm × 2 mm × 2 mm. Explicit spatial smoothing was not applied, as the overlapping multi-voxel information in adjacent searchlight spheres inherently produced sufficiently smooth images for analysis.

Before evaluating statistical significance of the Spearman correlations, the Shapiro-Wilk test for normality, as implemented in “scipy” (version 1.11.2), was applied to each voxel within the normalized Spearman correlation maps to evaluate the distribution of voxel values across participants. This aimed to determine the appropriateness of using SPM12 versus Statistical nonParametric Mapping (SnPM; https://www.nisox.org/Software/SnPM13)^93^ for subsequent second-level analyses. The results of the Shapiro-Wilk test were corrected for multiple comparisons using false discovery rate correction (p < 0.05, voxel-wise), utilizing the “statsmodels” package (version 0.14.0). It was revealed that with regard to the analysis based on the language RDM, only 0.40% of all non-NaN voxels diverged from a normal distribution, and in the case of the analysis based on the experience RDM, 0.49% of voxels did. Given the negligible proportion of deviating voxels, a one-sample t-test (in SPM12) was performed for each dataset to test voxel-wise whether the Spearman correlation coefficients were significantly different from zero across participants. After model estimation, a contrast testing for a positive main effect was specified. Additionally, two-sample t-tests were conducted to examine potential differences in voxel-wise Spearman correlation coefficients between graduate psychologists and undergraduate psychology students. One test focused on language-based semantic representational similarity, and the other on experience-based similarity. For each t-test, two separate t-contrasts investigated the contrast directions “psychologists > students” and “students > psychologists”, respectively. All statistical parametric maps generated from the above t-tests were thresholded at p < 0.001 (voxel-height), with an additional FWE correction applied at p < 0.05 at the cluster level.

We also performed a second set of RSA analyses that controlled for potential confounds such as low-level visual features, phonological features, and valence. Detailed methodological information is provided in the legend of Supplementary Figure S5.

MR data of the localizer tasks, which served to identify experiential brain circuits, were subjected to univariate fMRI activation analysis: Subject-wise, the realigned and coregistered EPIs from the localizer tasks (see above) were entered into a first-level GLM with a session-separated design matrix. The first session modeled the visual localizer, with a regressor representing the onsets and durations (24 s) of its respective task blocks. For the motor localizer, the onsets and durations (26 s) of both the left-hand and right-hand task blocks were amalgamated into one comprehensive regressor. In a similar vein, for the emotional-social scene observation localizer, the blocks of pleasant and unpleasant stimuli were merged, leading to the creation of two distinct regressors, representing the onsets and durations (23.8 s) of exposure to emotional-social and neutral images, respectively. The resulting boxcar functions were convolved with the canonical HRF. Additionally, the design matrix was augmented with the parameters stemming from realignment. Again, a high-pass filter (cutoff: 128 s) and an AR1 model were employed, and the individual skull-stripped T1 image was set as an explicit mask (without implicit masking). Upon model estimation, contrast images were generated to represent the main effect of each task condition relative to implicit baseline. Then, the forward deformation field, provided earlier by the CAT12 toolbox, was applied to all contrast images to implicitly normalize them to MNI space. This process also adjusted the voxel dimensions to 2 mm × 2 mm × 2 mm. The images were spatially smoothed, employing a Gaussian kernel of 6 mm full width at half maximum, before they were analyzed at the second level. For the visual and motor localizers, separate one-sample t-tests were conducted, whereas for the emotional-social scene observation localizer, a paired t-test was employed to compare the “emotional-social” condition against the “neutral” condition. For the resulting statistical parametric maps, a voxel-height threshold of *p* < 0.001 was applied, with cluster-level FWE correction set at *p* < 0.05. In addition to the parametric analyses, we calculated nonparametric permutation tests as comparison to provide an estimate of the robustness of our results with regard to the specific type of statistical approach (Supplementary Figure S6).

## Supplementary Information

Below is the link to the electronic supplementary material.

Supplementary Material 1

## Data Availability

Data and Python code have been deposited at Open Science Framework at https://osf.io/82utn/?view_only=d5b41f2e64e346968a56d2baffe52935.
