# Supplementary material for: Indirect experiential grounding: semantic similarity of abstract scientific concepts is reflected in activity patterns in visual and motor cortex
Source: Sci Rep. 2025 Dec 12;15:43814. doi: 10.1038/s41598-025-32189-2 (PMC12705760; doi:10.1038/s41598-025-32189-2)
Supplement: Supplementary file 1 — Supplementary Material 1 [file 41598_2025_32189_MOESM1_ESM.docx]

**Indirect experiential grounding: Semantic similarity of abstract scientific concepts is reflected in activity patterns in visual and motor cortex**

Martin Ulrich^1*^, Marcel Harpaintner^1^, Natalie M. Trumpp^1^, Alexander Berger^1^, Fritz Günther^2^, and
Markus Kiefer^1^

^1^Department of Psychiatry, Ulm University, Ulm, Germany.

^2^Department of Psychology, Humboldt University, Berlin, Germany.

*Correspondence: martin.ulrich@uni-ulm.de.

**Supplementary Information**

Contents:

- Supplementary Figure S1: Model Representational Dissimilarity Matrices
- Supplementary Figure S2: Results from Representational Similarity Analysis (RSA)
- Supplementary Figure S3: 3D renderings of the overlap of language-based RSA and localizer maps
- Supplementary Figure S4: 3D renderings of the overlap of experience-based RSA and localizer maps
- Supplementary Figure S5. Comparison of RSA results from two analysis pipelines with and without control for potential confounds
- Supplementary Figure S6. Comparison of RSA results based on parametric vs. nonparametric second-level inference
- Supplementary Table S1: Results from the visual localizer task
- Supplementary Table S2: Results from the motor localizer task
- Supplementary Table S3: Results from the emotional-social scene observation localizer task
- Supplementary Table S4: Tabular results from the exploratory, comparative analysis of language- and experience-based semantic representational similarity
- Supplementary Table S5: List of abstract psychological concepts and pseudowords
- Supplementary Table S6: Indices of IAPS pictures
- Python libraries and versions


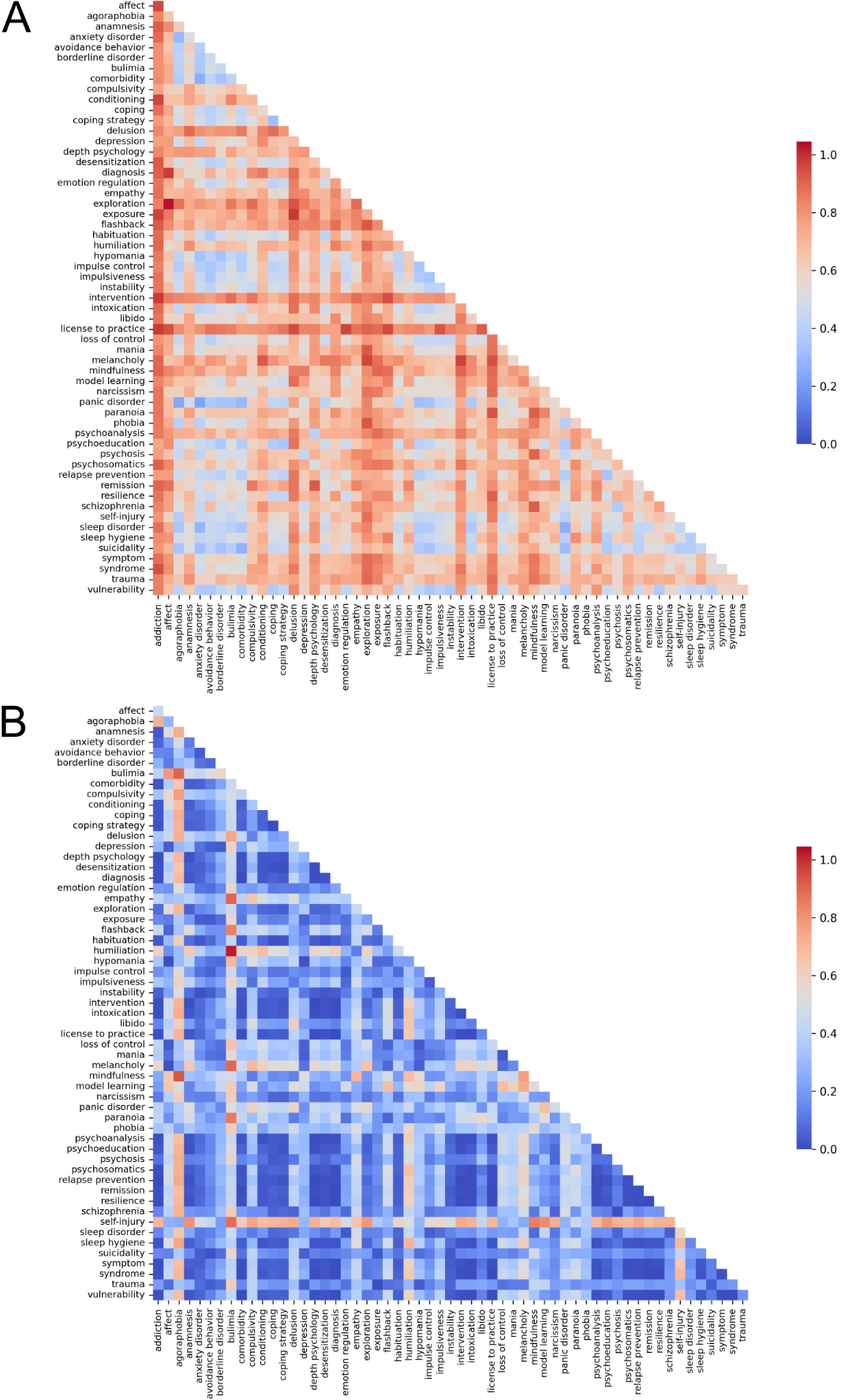


**Supplementary Figure S1.** Representational Dissimilarity Matrices illustrating the pairwise dissimilarities between the (analyzed) 58 concepts from the lexical decision task, **(A)** based on 400 features derived from a Continuous Bag of Words (CBOW) analysis of the German Wikipedia corpus “de_wiki”, downloaded from https://sites.google.com/site/fritzgntr/software-resources/semantic_spaces (last accessed July 25, 2023; see also Günther et al.^1^), and **(B)** based on the features from the property listing task (11 feature categories). Dissimilarity is quantified as 1 minus Pearson’s correlation coefficient. Warmer colors indicate greater dissimilarity between concepts within the semantic spaces.


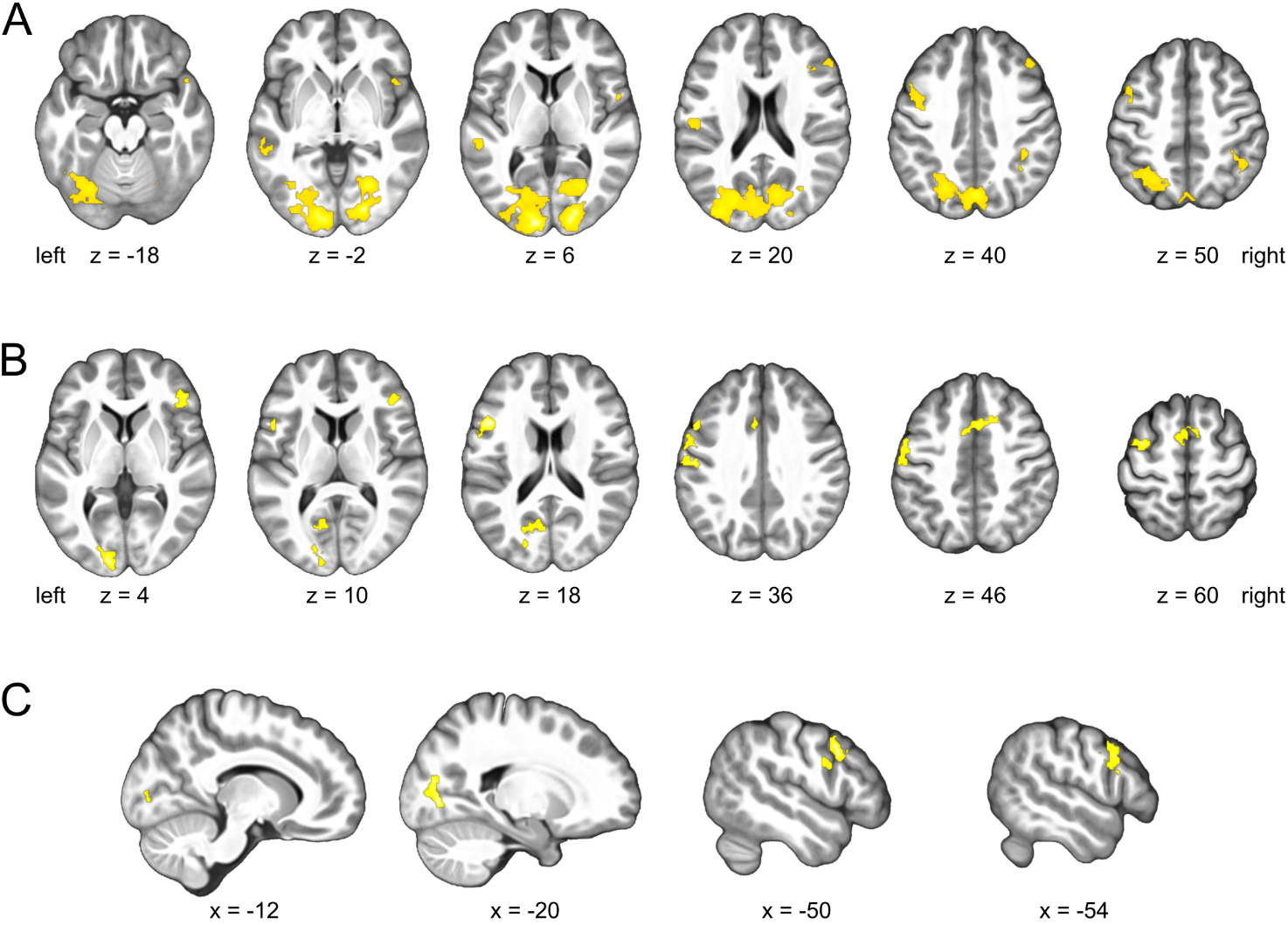


**Supplementary Figure S2.** Results from Representational Similarity Analysis (RSA). **(A)** Brain regions where the similarity between the language Representational Dissimilarity Matrix (RDM) and the searchlights’ neural RDMs was significant across all 51 participants, irrespective of their expertise. **(B)** Brain regions exhibiting significant representational similarity between the experience RDM and the searchlights’ neural RDMs (n = 51). **(C)** Clusters where the searchlights’ neural RDMs significantly Spearman-correlated with both the language and experience RDMs conjointly. This analysis was again conducted across all 51 participants, irrespective of their expertise. See also Supplementary Table S4. All statistical parametric maps were thresholded at p < 0.001, family-wise error rate (FWE)-corrected (p < 0.05) at the cluster level, and overlaid onto the mean normalized skull-stripped T1 image using MRIcroGL^2^. Coordinates refer to Montreal Neurological Institute (MNI) space.


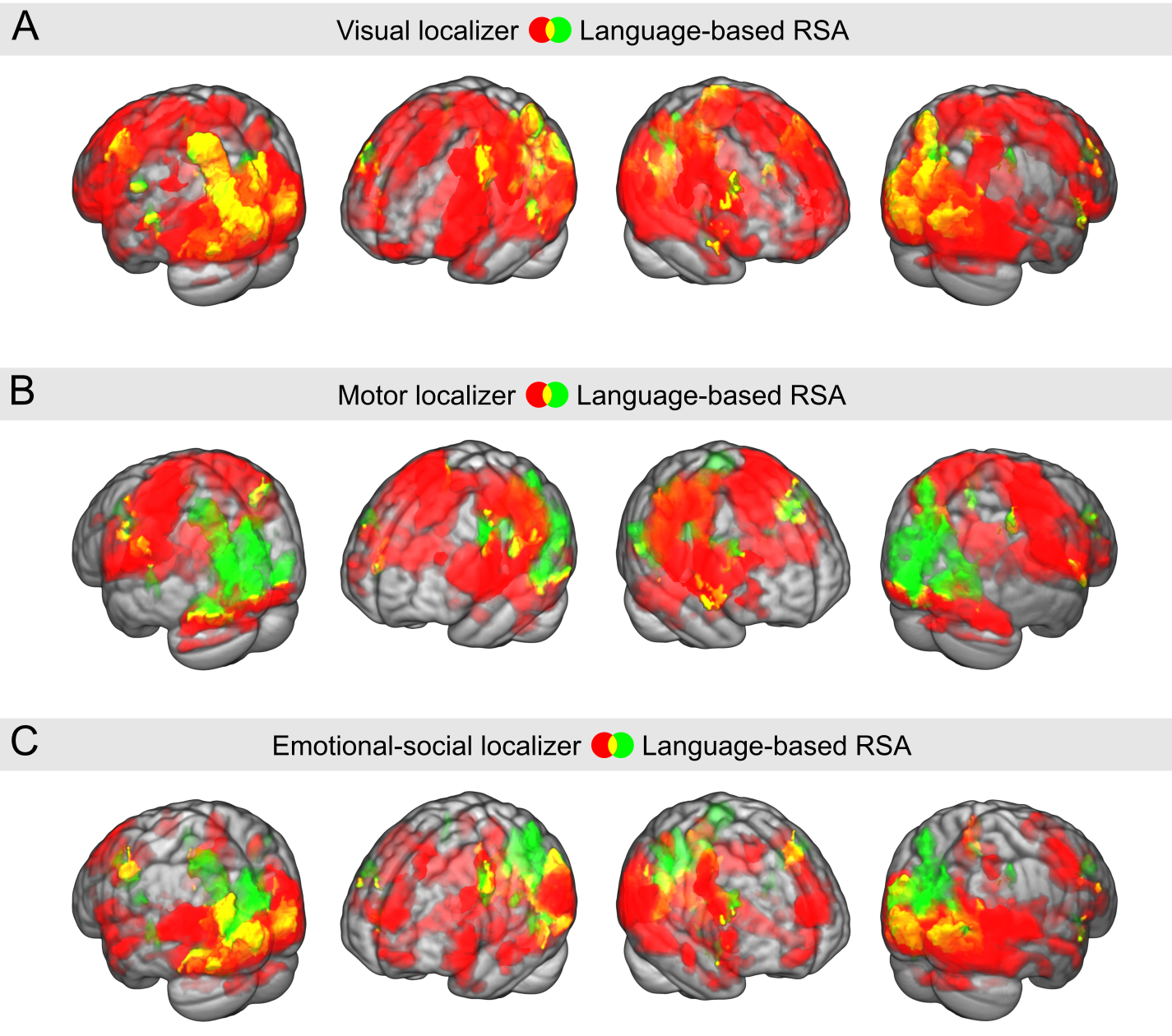


**Supplementary Figure S3.** Results from Representational Similarity Analysis (RSA) for the language-based semantic space (shown in green), alongside activation maps from the visual **(A)**, motor **(B)**, and emotional-social **(C)** localizer tasks (depicted in red). Areas of overlap appear in yellow. These semi-transparent 3D renderings offer a complementary visualization to the transversal slices shown in Figure 2 of the main article. All statistical parametric maps were thresholded at p < 0.001, family-wise error rate (FWE)-corrected (p < 0.05) at the cluster level, and overlaid onto the mean normalized skull-stripped T1 image using MRIcroGL^2^.


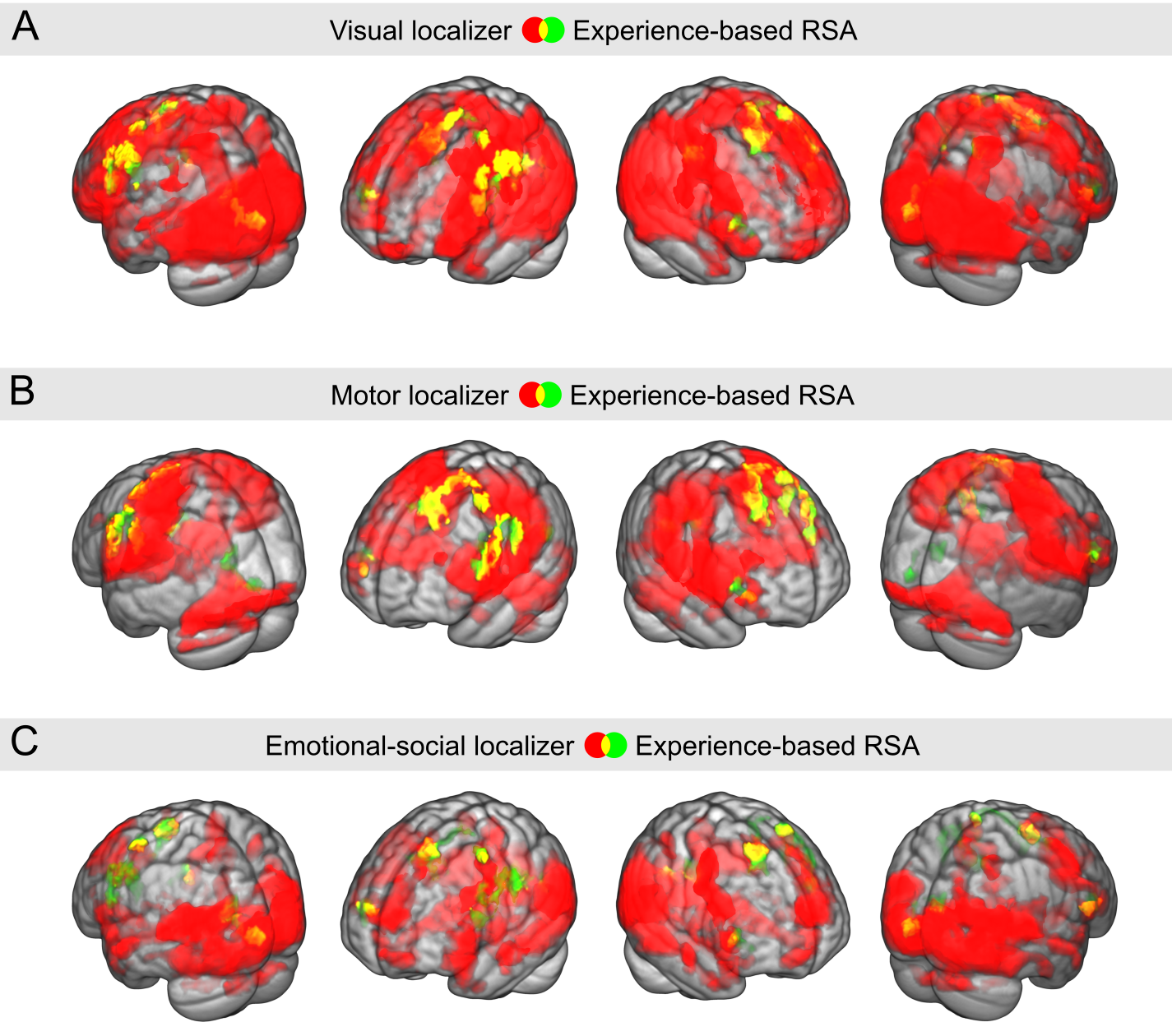


**Supplementary Figure S4.** Representational Similarity Analysis (RSA) results for the experience-based semantic space (shown in green), alongside activation maps from the visual **(A)**, motor **(B)**, and emotional-social **(C)** localizer tasks (depicted in red). Overlapping voxels are indicated in yellow. These semi-transparent 3D renderings again provide an additional perspective on the transversal slices presented in Figure 3 of the main article. All statistical parametric maps were thresholded at p < 0.001 (cluster-FWE-corrected, p < 0.05), and overlaid onto the mean normalized skull-stripped T1 image using MRIcroGL^2^.


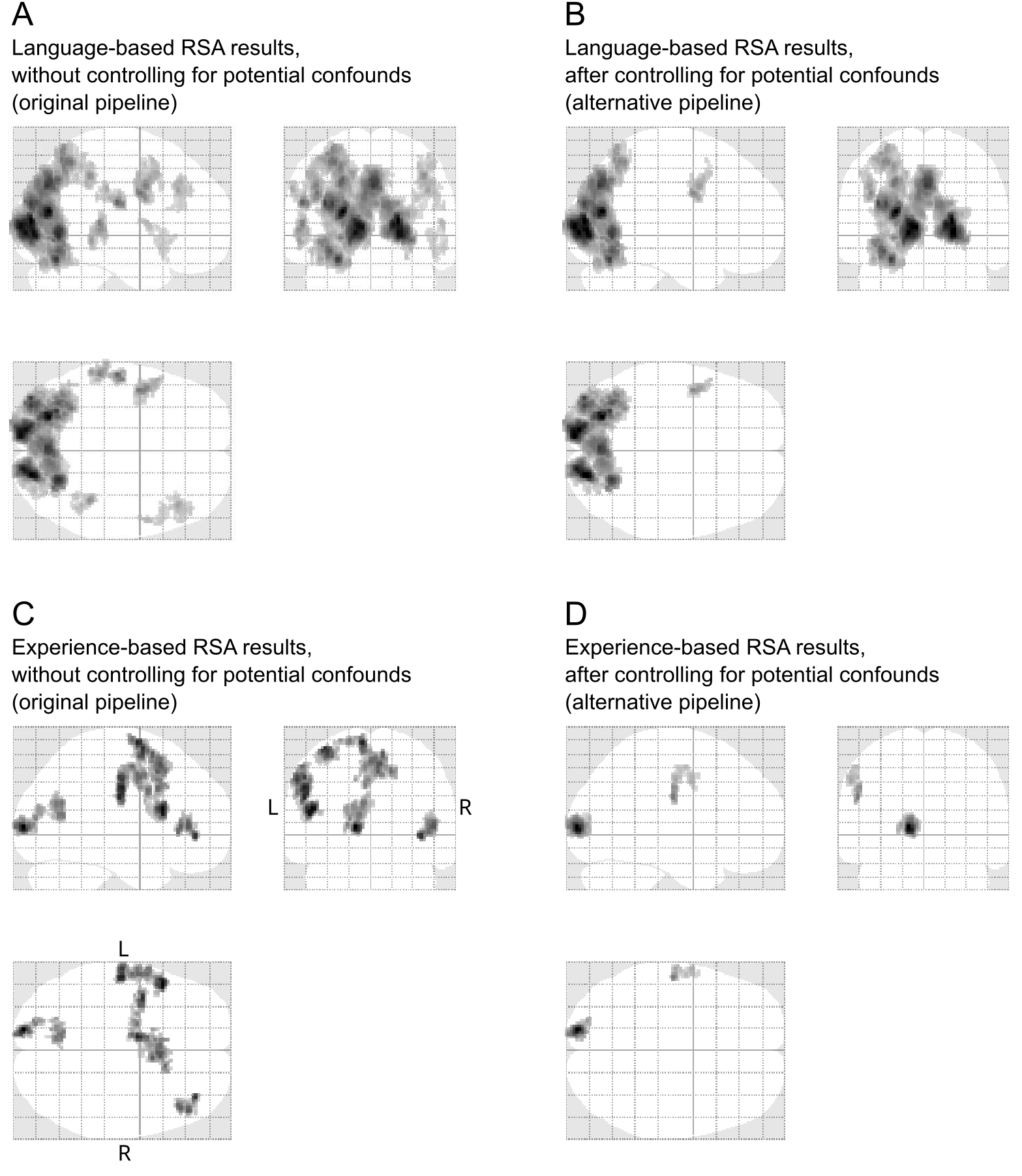


**Supplementary Figure S5.** Glass brain views illustrating Representational Similarity Analysis (RSA) results (p < 0.001, cluster-FWE-corrected, p < 0.05) from two analysis pipelines: one without controlling for potential confounds (**A** and **C**; original pipeline) and one controlling for low-level visual, phonological, and valence-related similarities (**B** and **D**; alternative pipeline). Results are shown separately for language-based RSA (**A** and **B**) and experience-based RSA (**C** and **D**). *Construction of control RDMs*: To account for alternative sources of similarity, four nuisance RDMs were created and regressed from the language- and experience-based RDMs prior to RSA: (1) Low-level visual RDM (based on Wang et al.^3^): Each word was rendered in the same font and size used in the main experiment as white text on a black canvas (≥ 500 × 200 px; final size matched to the maximal word bounds), then binarized, resized to 100 × 30 px, and vectorized. Pairwise dissimilarities were calculated as “1 - Pearson r”. (2) Phonological RDM (also based on ^3^): Words were manually transcribed into syllabified phonetic segments using the International Phonetic Alphabet. Each syllable was automatically decomposed into onset (initial consonants) and rhyme (vowel nucleus plus any coda consonants) components, using phonological criteria to identify German vowel nuclei (including monophthongs, diphthongs, and syllabic consonants). For each word, the union of all its syllabic onsets and rhymes was computed, yielding a set of sub-syllabic phonological units. Pairwise dissimilarities were calculated using a Jaccard-like distance metric: “distance(i, j) = 1 − (|shared units| / |union of units|)”. (3) Word-length RDM: Based on the absolute difference in character count between word pairs (Euclidean distance). (4) Valence RDM: Valence scores were obtained from a lexicon provided by Köper & Schulte im Walde^4^ (≈ 72 % verbatim matches). For out-of-vocabulary concepts, component words were averaged (e.g., “Emotion” and “Regulation” for “Emotionsregulation”) or substituted with a closely related lemma (e.g., “desensibilisieren” for “Desensibilisierung”). Pairwise dissimilarities were computed using the Euclidean distance metric. *Confound regression and RSA*: Each nuisance RDM was vectorized, Z-scaled, and simultaneously entered as a predictor in a linear model with either the language- or experience-based RDM as the dependent variable. The residuals, representing confound-removed RDMs, were submitted to the same RSA pipeline reported in the main text. *Outcome*: The alternative analysis pipeline, which accounted for low-level visual, phonological, and valence-related similarities, largely confirmed the originally observed RSA patterns. Thus, the language- and experience-based semantic representations reported in the main text are robust to these potential confounds. *Abbreviations*: L: left; R: right.


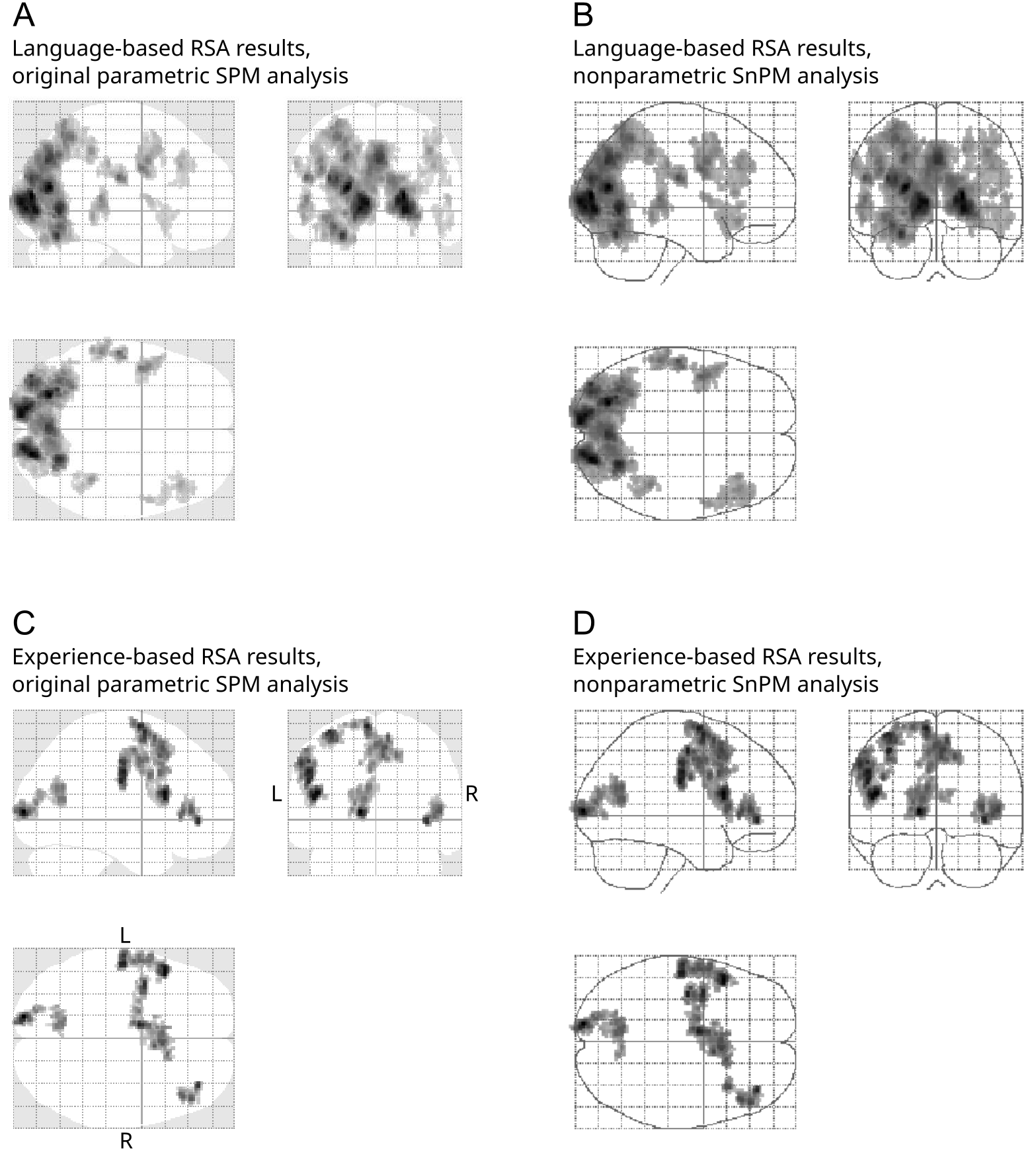


**Supplementary Figure S6.** Glass brain views comparing language- and experience-based RSA results from our original parametric SPM analyses (**A** and **C**) with its nonparametric counterparts (**B** and **D**) using Statistical nonParametric Mapping (SnPM13.1.09)^5^. Nonparametric inference was based on 10000 permutations, with cluster-level FWE correction (p < 0.05) following a voxel-wise cluster-forming threshold of p < 0.001.

**Supplementary Table S1.** Results from the visual localizer task, listing brain regions with significantly greater activation for viewing pictures of animate and inanimate objects compared to fixation cross, based on data from the entire sample of 51 participants. Voxel-height threshold: p < 0.001, family-wise error rate (FWE)-corrected (p < 0.05) at the cluster level. Coordinates are given in Montreal Neurological Institute space. Abbreviations: L: left; R: right.

| Brain region | Cluster size | Peak voxel | | | |
| --- | --- | --- | --- | --- | --- |
|  | (in voxels) | x | y | z | z-score |
| Inferior occipital gyrus (L) | 68209 | -38 | -80 | -12 | 11.50 |
| Fusiform gyrus (L) |  | -28 | -68 | -10 | 11.44 |
| Lingual gyrus (R) |  | 18 | -90 | -6 | 11.26 |
| Fusiform gyrus (L) |  | -32 | -46 | -18 | 11.21 |
| Inferior occipital gyrus (R) |  | 38 | -68 | -12 | 11.18 |
| Inferior occipital gyrus (R) |  | 44 | -82 | -2 | 11.15 |
| Fusiform gyrus (R) |  | 30 | -82 | -8 | 11.13 |
| Lingual gyrus (L) |  | -18 | -86 | -8 | 11.02 |
| Inferior occipital gyrus (L) |  | -14 | -94 | -8 | 10.78 |
| Fusiform gyrus (R) |  | 34 | -46 | -18 | 10.49 |
| Hippocampus (L) |  | -20 | -32 | -2 | 10.34 |
| Middle occipital gyrus (L) |  | -30 | -94 | 10 | 10.28 |
| Superior occipital gyrus (R) |  | 22 | -98 | 12 | 10.01 |
| Middle occipital gyrus (L) |  | -28 | -84 | 20 | 9.93 |
| Hippocampus (R) |  | 22 | -28 | -2 | 9.71 |
| Middle occipital gyrus (L) |  | -26 | -92 | 20 | 9.64 |
| Lingual gyrus (R) |  | 20 | -56 | 2 | 9.53 |
| Calcarine (R) |  | 10 | -68 | 16 | 9.38 |
| Middle occipital gyrus (R) |  | 30 | -78 | 18 | 9.36 |
| Calcarine (L) |  | -6 | -72 | 16 | 9.26 |
| Lingual gyrus (L) |  | -14 | -58 | 0 | 9.01 |
| Inferior frontal gyrus, orbital part (L) |  | -34 | 34 | -18 | 8.10 |
| Superior frontal gyrus (L) |  | -14 | 54 | 40 | 8.00 |
| Inferior temporal gyrus (L) |  | -44 | -46 | -12 | 7.99 |
| Fusiform gyrus (L) |  | -32 | -4 | -34 | 7.89 |
| Superior parietal lobule (L) |  | -28 | -58 | 58 | 7.80 |
| Paracentral lobule (L) |  | -10 | -30 | 70 | 7.75 |
| Fusiform gyrus (R) |  | 30 | -2 | -38 | 7.58 |
| Precentral gyrus (R) |  | 50 | 0 | 54 | 7.50 |
| Precentral gyrus (L) |  | -50 | -10 | 50 | 7.50 |
| Supplementary motor area (R) |  | 6 | 0 | 64 | 7.34 |
| Amygdala (L) |  | -30 | 0 | -20 | 7.27 |
| Inferior frontal gyrus, triangular part (L) |  | -42 | 26 | 0 | 7.25 |
| Precentral gyrus (L) |  | -46 | 0 | 38 | 7.20 |
| Inferior frontal gyrus, triangular part (L) |  | -44 | 28 | 18 | 6.94 |
| Superior parietal lobule (R) |  | 26 | -56 | 52 | 6.88 |
| Supplementary motor area (L) |  | -6 | 8 | 56 | 6.83 |
| Precentral gyrus (L) |  | -38 | -24 | 56 | 6.79 |
| Postcentral gyrus (R) |  | 68 | -4 | 22 | 6.75 |
| Middle temporal gyrus (R) |  | 46 | -54 | 6 | 6.61 |
| Inferior frontal gyrus, orbital part (R) |  | 32 | 36 | -14 | 6.54 |
| Superior temporal gyrus (R) |  | 48 | -36 | 10 | 6.46 |
| Middle temporal gyrus (L) |  | -48 | -46 | 16 | 6.46 |
| Inferior frontal gyrus, triangular part (R) |  | 58 | 32 | 18 | 6.42 |
| Inferior frontal gyrus, opercular part (L) |  | -40 | 10 | 28 | 6.30 |
| Cuneus (R) |  | 10 | -82 | 34 | 6.28 |
| Inferior frontal gyrus, opercular part (R) |  | 38 | 8 | 30 | 6.27 |
| Amygdala (R) |  | 28 | 0 | -20 | 6.23 |
| Postcentral gyrus (L) |  | -62 | -8 | 36 | 6.22 |
| Precentral gyrus (R) |  | 32 | -22 | 54 | 5.93 |
| Temporal pole (L) |  | -46 | 20 | -26 | 5.78 |
| Postcentral gyrus (L) |  | -42 | -34 | 60 | 5.77 |
| Middle temporal gyrus (L) |  | -52 | -24 | -4 | 5.57 |
| Postcentral gyrus (R) |  | 38 | -32 | 62 | 5.31 |
| Temporal pole (R) |  | 48 | 20 | -20 | 4.51 |
| Insula (R) |  | 32 | 28 | 6 | 4.46 |
| Cerebellum (L) | 136 | -28 | -66 | -46 | 6.46 |
| Superior temporal gyrus (L) | 119 | -42 | -36 | 26 | 5.18 |
| Insula (L) | 252 | -36 | -8 | 16 | 4.92 |
| Putamen (L) |  | -22 | -2 | 8 | 4.09 |

**Supplementary Table S2.** Brain regions showing significant activation during the motor localizer task, contrasting hand movements against visual fixation in all 51 participants. Voxel-height threshold: p < 0.001, cluster-FWE-corrected (p < 0.05). Abbreviations are consistent with those in Supplementary Table S1.

| Brain region | Cluster size | Peak voxel | | | |
| --- | --- | --- | --- | --- | --- |
|  | (in voxels) | x | y | z | z-score |
| Cerebellum (L) | 40682 | -22 | -54 | -22 | 10.13 |
| Precentral gyrus (R) |  | 42 | -22 | 56 | 9.74 |
| Cerebellum (L) |  | -28 | -48 | -28 | 9.48 |
| Supplementary motor area (L) |  | -4 | -4 | 54 | 9.39 |
| Precentral gyrus (R) |  | 40 | -12 | 60 | 9.04 |
| Cerebellum (R) |  | 34 | -50 | -30 | 8.85 |
| Insula (R) |  | 46 | 4 | 2 | 8.82 |
| Cerebellum (R) |  | 4 | -64 | -14 | 8.80 |
| Supramarginal gyrus (L) |  | -50 | -24 | 18 | 8.74 |
| Cerebellum (R) |  | 14 | -52 | -20 | 8.69 |
| Supplementary motor area (R) |  | 6 | 0 | 48 | 8.66 |
| Putamen (R) |  | 26 | -2 | 4 | 8.48 |
| Supplementary motor area (R) |  | 6 | -2 | 66 | 8.41 |
| Thalamus (R) |  | 14 | -16 | 4 | 8.33 |
| Cerebellum (L) |  | -22 | -60 | -48 | 8.32 |
| Putamen (L) |  | -26 | -4 | 6 | 8.25 |
| Thalamus (L) |  | -12 | -20 | 6 | 8.18 |
| Rolandic operculum (R) |  | 50 | -20 | 18 | 7.74 |
| Precentral gyrus (L) |  | -40 | -16 | 56 | 7.69 |
| Postcentral gyrus (R) |  | 54 | -20 | 46 | 7.67 |
| Middle cingulate cortex (L) |  | -8 | 4 | 36 | 7.41 |
| Insula (L) |  | -36 | 2 | 2 | 7.34 |
| Precentral gyrus (R) |  | 60 | 10 | 18 | 7.07 |
| Supramarginal gyrus (L) |  | -58 | -24 | 40 | 7.00 |
| Postcentral gyrus (L) |  | -44 | -26 | 48 | 6.84 |
| Precentral gyrus (L) |  | -58 | 6 | 24 | 6.80 |
| Precentral gyrus (L) |  | -32 | -10 | 66 | 6.55 |
| Insula (L) |  | -30 | 18 | 6 | 6.46 |
| Precentral gyrus (R) |  | 44 | -2 | 58 | 6.45 |
| Superior temporal gyrus (R) |  | 58 | -34 | 22 | 6.34 |
| Putamen (R) |  | 20 | 14 | 2 | 5.94 |
| Inferior parietal lobule (L) |  | -50 | -36 | 54 | 5.59 |
| Calcarine (L) |  | -14 | -104 | -8 | 5.30 |
| Lingual gyrus (R) |  | 22 | -92 | -6 | 5.06 |
| Precentral gyrus (R) |  | 54 | 8 | 38 | 4.96 |
| Inferior parietal lobule (R) |  | 42 | -42 | 46 | 4.30 |
| Middle cingulate cortex (R) |  | 2 | 18 | 36 | 3.56 |
| Middle frontal gyrus (R) | 989 | 36 | 40 | 26 | 4.72 |
| Inferior frontal gyrus, orbital part (R) |  | 42 | 44 | -6 | 4.55 |
| Middle frontal gyrus (R) |  | 42 | 48 | 6 | 4.33 |
| Middle frontal gyrus (L) | 147 | -38 | 40 | 26 | 4.66 |

**Supplementary Table S3.** Brain regions with significantly greater activation for emotional-social versus neutral pictures during the emotional-social scene observation localizer task (n = 51). Voxel-height threshold: p < 0.001, cluster-FWE-corrected (p < 0.05). Abbreviations are consistent with those in Supplementary Table S1.

| Brain region | Cluster size | Peak voxel | | | |
| --- | --- | --- | --- | --- | --- |
|  | (in voxels) | x | y | z | z-score |
| Calcarine (R) | 31140 | 10 | -98 | 8 | 9.58 |
| Middle temporal gyrus (R) |  | 52 | -72 | 6 | 9.42 |
| Middle occipital gyrus (L) |  | -48 | -80 | 4 | 9.02 |
| Fusiform gyrus (R) |  | 42 | -50 | -22 | 9.00 |
| Fusiform gyrus (L) |  | -42 | -60 | -16 | 8.88 |
| Fusiform gyrus (R) |  | 40 | -64 | -18 | 8.73 |
| Middle occipital gyrus (L) |  | -12 | -102 | 6 | 8.71 |
| Middle temporal gyrus (R) |  | 44 | -58 | 14 | 8.66 |
| Fusiform gyrus (L) |  | -40 | -46 | -18 | 8.41 |
| Hippocampus (L) |  | -20 | -6 | -14 | 8.29 |
| Cerebellum (R) |  | 8 | -80 | -40 | 8.06 |
| Inferior occipital gyrus (R) |  | 36 | -84 | -8 | 7.57 |
| Fusiform gyrus (L) |  | -36 | -74 | -14 | 7.56 |
| Middle temporal gyrus (L) |  | -48 | -68 | 16 | 7.53 |
| Hippocampus (R) |  | 20 | -6 | -14 | 7.14 |
| Middle occipital gyrus (L) |  | -42 | -86 | -4 | 6.99 |
| Superior temporal gyrus (R) |  | 50 | -40 | 16 | 6.94 |
| Middle occipital gyrus (R) |  | 28 | -90 | 10 | 6.77 |
| Middle temporal gyrus (L) |  | -60 | -52 | 10 | 6.36 |
| Hippocampus (L) |  | -20 | -32 | -4 | 6.34 |
| Lingual gyrus (L) |  | -16 | -88 | -16 | 6.31 |
| Precuneus (L) |  | -2 | -52 | 12 | 6.22 |
| Amygdala (R) |  | 32 | 6 | -20 | 5.65 |
| Temporal pole (R) |  | 40 | 20 | -26 | 5.64 |
| Lingual gyrus (R) |  | 20 | -58 | -8 | 5.63 |
| Middle occipital gyrus (R) |  | 38 | -80 | 24 | 5.61 |
| Hippocampus (L) |  | -32 | -26 | -10 | 5.60 |
| Calcarine (L) |  | -6 | -74 | 8 | 5.57 |
| Fusiform gyrus (R) |  | 34 | -4 | -38 | 5.56 |
| Inferior frontal gyrus, triangular part (L) |  | -44 | 28 | 2 | 5.37 |
| Hippocampus (R) |  | 32 | -18 | -12 | 5.25 |
| Inferior frontal gyrus, orbital part (R) |  | 32 | 30 | -18 | 5.17 |
| Temporal pole (L) |  | -44 | 20 | -28 | 4.99 |
| Precuneus (R) |  | 6 | -50 | 52 | 4.98 |
| Putamen (L) |  | -30 | 2 | -6 | 4.93 |
| Thalamus (R) |  | 14 | -18 | 4 | 4.17 |
| Medial superior frontal gyrus (L) | 2278 | -6 | 64 | 30 | 6.89 |
| Medial superior frontal gyrus (L) |  | -6 | 52 | 30 | 5.85 |
| Medial superior frontal gyrus (L) |  | 4 | 62 | 14 | 5.80 |
| Superior frontal gyrus (L) |  | -22 | 62 | 28 | 4.92 |
| Anterior cingulate cortex (L) |  | -12 | 46 | 10 | 4.06 |
| Middle temporal gyrus (R) | 267 | 52 | -6 | -16 | 6.71 |
| Gyrus rectus (L) | 535 | -2 | 42 | -22 | 6.27 |
| Medial orbital frontal gyrus (L) |  | -4 | 66 | -8 | 3.94 |
| Precentral gyrus (L) | 913 | -36 | -4 | 52 | 6.21 |
| Inferior frontal gyrus, opercular part (L) |  | -40 | 10 | 30 | 4.71 |
| Precentral gyrus (L) |  | -48 | 0 | 38 | 4.29 |
| Middle temporal gyrus (L) | 449 | -54 | -10 | -12 | 5.96 |
| Middle temporal gyrus (L) |  | -64 | -12 | -16 | 5.16 |
| Middle temporal gyrus (L) |  | -62 | 0 | -16 | 4.57 |
| Precentral gyrus (R) | 2236 | 42 | -2 | 48 | 5.90 |
| Precentral gyrus (R) |  | 38 | 6 | 32 | 5.69 |
| Inferior frontal gyrus, triangular part (R) |  | 42 | 22 | 24 | 5.41 |
| Precentral gyrus (R) |  | 52 | 8 | 46 | 5.38 |
| Inferior frontal gyrus, triangular part (R) |  | 46 | 28 | -2 | 5.25 |
| Inferior frontal gyrus, triangular part (R) |  | 56 | 38 | 12 | 5.17 |
| Inferior parietal lobule (R) | 345 | 30 | -52 | 50 | 5.69 |
| Cerebellum (R) | 136 | 18 | -42 | -44 | 5.54 |
| Cerebellum (L) | 544 | -20 | -44 | -42 | 5.43 |
| Cerebellum (R) |  | 2 | -52 | -36 | 4.87 |
| Supplementary motor area (R) | 422 | 6 | 10 | 56 | 5.17 |
| Thalamus (L) | 140 | -6 | -10 | 8 | 4.01 |

**Supplementary Table S4.** Clusters where the searchlights’ neural Representational Dissimilarity Matrices (RDM) significantly Spearman-correlated with both the language and experience RDMs conjointly. Coordinates are given in Montreal Neurological Institute space. See also Supplementary Figure S2C.

| Brain region | Cluster size | Peak voxel | | | |
| --- | --- | --- | --- | --- | --- |
|  | (in voxels) | x | y | z | z-score |
| Left middle frontal gyrus | 190 | -50 | 14 | 36 | 4.27 |
| Left precentral sulcus |  | -54 | 8 | 42 | 3.74 |
| Left inferior frontal gyrus, opercular part |  | -54 | 10 | 26 | 3.53 |
| Left precentral gyrus |  | -48 | 2 | 26 | 3.46 |
| Left lingual gyrus | 153 | -22 | -78 | -2 | 4.12 |
| Left superior occipital gyrus |  | -22 | -80 | 6 | 3.95 |
| Left calcarine |  | -20 | -80 | 14 | 3.90 |

**Supplementary Table S5.** List of abstract psychological concepts and pseudowords employed in the lexical decision task.

| Meaningful words | | Pseudowords |
| --- | --- | --- |
| German (original) | English (translation) |  |
| Achtsamkeit  Affekt  Agoraphobie  Anamnese  Angststörung  Approbation  Belohnungsaufschub  Bewältigungsstrategie  Borderline-Störung  Bulimie  Coping  Depression  Desensibilisierung  Diagnose  Emotionsregulation  Empathie  Exploration  Exposition  Flashback  Habituation  Hypomanie  Imaginationsübung  Impulsivität  Impulskontrolle  Intervention  Intoxikation  Katastrophisierung  Komorbidität  Konditionierung  Kontrollverlust  Kränkung  Labilität  Libido  Manie  Melancholie  Modelllernen  Narzissmus  Panikstörung  Paranoia  Persönlichkeitsakzentuierung  Phobie  Psychoanalyse  Psychoedukation  Psychose  Psychosomatik  Remission  Resilienz  Rückfallprophylaxe  Schizophrenie  Schlafhygiene  Schlafstörung  Selbstverletzung  Sicherheitsverhalten  Sucht  Suizidalität  Symptom  Syndrom  Tiefenpsychologie  Trauma  Verhaltensexperiment  Vermeidungsverhalten  Vulnerabilität  Wahn  Zwanghaftigkeit | mindfulness  affect  agoraphobia  anamnesis  anxiety disorder  license to practice  delayed reward  coping strategy  borderline disorder  bulimia  coping  depression  desensitization  diagnosis  emotion regulation  empathy  exploration  exposure  flashback  habituation  hypomania  imagination exercise  impulsiveness  impulse control  intervention  intoxication  catastrophization  comorbidity  conditioning  loss of control  humiliation  instability  libido  mania  melancholy  model learning  narcissism  panic disorder  paranoia  personality accentuation  phobia  psychoanalysis  psychoeducation  psychosis  psychosomatics  remission  resilience  relapse prevention  schizophrenia  sleep hygiene  sleep disorder  self-injury  safety behavior  addiction  suicidality  symptom  syndrome  depth psychology  trauma  behavioral experiment  avoidance behavior  vulnerability  delusion  compulsivity | Äberkomkensation  Anpassengsstökung  Attmibutien  Attributeonsskil  Augosuggastion  Avargion  Bikdungsstärung  Cospitelismus  Daprization  Dassorialität  Delkstürung  Derualisapion  Diskrikinationstrainung  Drößenodee  Dunkschera  Dylpherie  Dynthymoa  Exminktuon  Extriverlion  Ezathie  Funptionsniveiu  Gedinkenstomp  Geparanting  Gonusstraiping  Greppenkogäsion  Gröbelzfang  Hykervigolanz  Hypurvenmilation  Iggranation  Imenorrtoe  Itiolonie  Izeenflecht  Kolfabilation  Kränwburkeit  Kürperdyskorphie  Kürperscheka  Lerzneurase  Masichigmus  Mitakorge  Morganlief  Nogativsymftom  Ogitiertjeit  Onhebonie  Posiwivsymptem  Prödisnosition  Psychahytiene  Qrodrimal  Ralionalusierung  Razibiv  Regiwität  Ruscrigting  Salbstsicherweit  Schazotymie  Seggeslion  Sissimelation  Somutikierung  Sorgunrette  Stöhlearkeit  Unankusmus  Untrogekt  Vogitanz  Wesidualsymptem  Zwingsimruls  Zymlothymua |

**Supplementary Table S6.** Indices of IAPS^6^ pictures employed in the emotional-social localizer task.

| Pleasant pictures | Unpleasant pictures | Neutral pictures,  subset 1 | Neutral pictures,  subset 2 |
| --- | --- | --- | --- |
| 1440  1460  1920  2040  2050  2057  2071  2080  2091  2216  2340  2550  5621  5830  8080  8190  8200  8370  8496  8499 | 2095  2205  2276  2703  2800  2981  3180  3230  3300  6313  6825  9220  9254  9265  9320  9561  9800  9810  9910  9921 | 2102  2396  2516  5531  7002  7010  7020  7035  7041  7050  7056  7059  7090  7160  7161  7170  7179  7185  7207  7640 | 1935  2214  2397  2880  2890  5510  6150  7000  7004  7009  7034  7043  7055  7187  7233  7235  7484  7487  7950  9070 |

***Python libraries and versions***

We employed “rsatoolbox” (version 0.1.3) under Python (version 3.11.4) to conduct Representational Similarity Analysis. Below is a list of relevant Python packages and their specific versions used, including sub-dependencies, as determined by “pipdeptree” (version 2.15.1):

rsatoolbox==0.1.3

├── h5py [required: Any, installed: 3.9.0]

│ └── numpy [required: >=1.17.3, installed: 1.25.2]

├── joblib [required: Any, installed: 1.3.2]

├── matplotlib [required: Any, installed: 3.7.2]

│ ├── contourpy [required: >=1.0.1, installed: 1.1.0]

│ │ └── numpy [required: >=1.16, installed: 1.25.2]

│ ├── cycler [required: >=0.10, installed: 0.11.0]

│ ├── fonttools [required: >=4.22.0, installed: 4.42.1]

│ ├── kiwisolver [required: >=1.0.1, installed: 1.4.4]

│ ├── numpy [required: >=1.20, installed: 1.25.2]

│ ├── packaging [required: >=20.0, installed: 23.0]

│ ├── Pillow [required: >=6.2.0, installed: 10.0.0]

│ ├── pyparsing [required: >=2.3.1,<3.1, installed: 3.0.9]

│ └── python-dateutil [required: >=2.7, installed: 2.8.2]

│ └── six [required: >=1.5, installed: 1.16.0]

├── numpy [required: >=1.21.2, installed: 1.25.2]

├── pandas [required: Any, installed: 2.0.3]

│ ├── numpy [required: >=1.21.0, installed: 1.25.2]

│ ├── numpy [required: >=1.23.2, installed: 1.25.2]

│ ├── python-dateutil [required: >=2.8.2, installed: 2.8.2]

│ │ └── six [required: >=1.5, installed: 1.16.0]

│ ├── pytz [required: >=2020.1, installed: 2022.7]

│ └── tzdata [required: >=2022.1, installed: 2023.3]

├── scikit-image [required: Any, installed: 0.21.0]

│ ├── imageio [required: >=2.27, installed: 2.31.1]

│ │ ├── numpy [required: Any, installed: 1.25.2]

│ │ └── Pillow [required: >=8.3.2, installed: 10.0.0]

│ ├── lazy-loader [required: >=0.2, installed: 0.3]

│ ├── networkx [required: >=2.8, installed: 3.1]

│ ├── numpy [required: >=1.21.1, installed: 1.25.2]

│ ├── packaging [required: >=21, installed: 23.0]

│ ├── Pillow [required: >=9.0.1, installed: 10.0.0]

│ ├── PyWavelets [required: >=1.1.1, installed: 1.4.1]

│ │ └── numpy [required: >=1.17.3, installed: 1.25.2]

│ ├── scipy [required: >=1.8, installed: 1.11.2]

│ │ └── numpy [required: >=1.21.6,<1.28.0, installed: 1.25.2]

│ └── tifffile [required: >=2022.8.12, installed: 2023.8.12]

│ └── numpy [required: Any, installed: 1.25.2]

├── scikit-learn [required: Any, installed: 1.3.0]

│ ├── joblib [required: >=1.1.1, installed: 1.3.2]

│ ├── numpy [required: >=1.17.3, installed: 1.25.2]

│ ├── scipy [required: >=1.5.0, installed: 1.11.2]

│ │ └── numpy [required: >=1.21.6,<1.28.0, installed: 1.25.2]

│ └── threadpoolctl [required: >=2.0.0, installed: 3.2.0]

├── scipy [required: Any, installed: 1.11.2]

│ └── numpy [required: >=1.21.6,<1.28.0, installed: 1.25.2]

└── tqdm [required: Any, installed: 4.66.1]

└── colorama [required: Any, installed: 0.4.6]

nibabel==5.1.0

├── numpy [required: >=1.19, installed: 1.25.2]

└── packaging [required: >=17, installed: 23.0]

nilearn==0.10.1

├── joblib [required: >=1.0.0, installed: 1.3.2]

├── lxml [required: Any, installed: 4.9.2]

├── nibabel [required: >=3.2.0, installed: 5.1.0]

│ ├── numpy [required: >=1.19, installed: 1.25.2]

│ └── packaging [required: >=17, installed: 23.0]

├── numpy [required: >=1.19.0, installed: 1.25.2]

├── packaging [required: Any, installed: 23.0]

├── pandas [required: >=1.1.5, installed: 2.0.3]

│ ├── numpy [required: >=1.21.0, installed: 1.25.2]

│ ├── numpy [required: >=1.23.2, installed: 1.25.2]

│ ├── python-dateutil [required: >=2.8.2, installed: 2.8.2]

│ │ └── six [required: >=1.5, installed: 1.16.0]

│ ├── pytz [required: >=2020.1, installed: 2022.7]

│ └── tzdata [required: >=2022.1, installed: 2023.3]

├── requests [required: >=2.25.0, installed: 2.31.0]

│ ├── certifi [required: >=2017.4.17, installed: 2023.7.22]

│ ├── charset-normalizer [required: >=2,<4, installed: 2.0.4]

│ ├── idna [required: >=2.5,<4, installed: 3.4]

│ └── urllib3 [required: >=1.21.1,<3, installed: 1.26.16]

├── scikit-learn [required: >=1.0.0, installed: 1.3.0]

│ ├── joblib [required: >=1.1.1, installed: 1.3.2]

│ ├── numpy [required: >=1.17.3, installed: 1.25.2]

│ ├── scipy [required: >=1.5.0, installed: 1.11.2]

│ │ └── numpy [required: >=1.21.6,<1.28.0, installed: 1.25.2]

│ └── threadpoolctl [required: >=2.0.0, installed: 3.2.0]

└── scipy [required: >=1.6.0, installed: 1.11.2]

└── numpy [required: >=1.21.6,<1.28.0, installed: 1.25.2]

scipy==1.11.2

└── numpy [required: >=1.21.6,<1.28.0, installed: 1.25.2]

statsmodels==0.14.0

├── numpy [required: >=1.18, installed: 1.25.2]

├── packaging [required: >=21.3, installed: 23.0]

├── pandas [required: >=1.0, installed: 2.0.3]

│ ├── numpy [required: >=1.21.0, installed: 1.25.2]

│ ├── numpy [required: >=1.23.2, installed: 1.25.2]

│ ├── python-dateutil [required: >=2.8.2, installed: 2.8.2]

│ │ └── six [required: >=1.5, installed: 1.16.0]

│ ├── pytz [required: >=2020.1, installed: 2022.7]

│ └── tzdata [required: >=2022.1, installed: 2023.3]

├── patsy [required: >=0.5.2, installed: 0.5.3]

│ ├── numpy [required: >=1.4, installed: 1.25.2]

│ └── six [required: Any, installed: 1.16.0]

├── scipy [required: >=1.4,!=1.9.2, installed: 1.11.2]

│ └── numpy [required: >=1.21.6,<1.28.0, installed: 1.25.2]

└── scipy [required: >=1.4,!=1.9.2, installed: 1.11.2]

└── numpy [required: >=1.21.6,<1.28.0, installed: 1.25.2]

**References**

1. Günther, F., Dudschig, C. & Kaup, B. LSAfun - An R package for computations based on Latent Semantic Analysis. *Behav. Res. Methods* **47**, 930-944 (2015).

2. Rorden, C. & Brett, M. Stereotaxic display of brain lesions. *Behav. Neurol.* **12**, 191-200 (2000).

3. Wang, X., Wang, B. & Bi, Y. Early language exposure affects neural mechanisms of semantic representations. *eLife* **12** (2023).

4. Köper, M. & Schulte im Walde, S. Automatically generated affective norms of abstractness, arousal, imageability and valence for 350 000 German lemmas. *LREC 2016 - Tenth International Conference on Language Resources and Evaluation*, 2595-2598 (2016).

5. Nichols, T.E. & Holmes, A.P. Nonparametric permutation tests for functional neuroimaging: a primer with examples. *Hum. Brain Mapp.* **15**, 1-25 (2002).

6. Lang, P.J., Bradley, M.M. & Cuthbert, B.N. *International Affective Picture System (IAPS): Instruction manual and affective ratings, Technical Report A-8* (The Center for Research in Psychophysiology, University of Florida, Gainesville, 2008).
